# Hepatic interleukin‐1 receptor type 1 signalling regulates insulin sensitivity in the early phases of nonalcoholic fatty liver disease

**DOI:** 10.1002/ctm2.1048

**Published:** 2022-09-13

**Authors:** Nadine Gehrke, Lea J. Hofmann, Beate K. Straub, Frank Rühle, Ari Waisman, Peter R. Galle, Jörn M. Schattenberg

**Affiliations:** ^1^ I. Department of Medicine University Medical Center of the Johannes Gutenberg University Mainz Mainz 55131 Germany; ^2^ Institute of Pathology University Medical Center of the Johannes Gutenberg University Mainz Mainz Germany; ^3^ Bioinformatics Core Facility Institute of Molecular Biology (IMB) Mainz Germany; ^4^ Institute for Molecular Medicine University Medical Center of the Johannes Gutenberg University Mainz Mainz Germany; ^5^ Research Center for Immunotherapy University Medical Center of the Johannes Gutenberg University Mainz Mainz Germany

**Keywords:** cirrhosis, hepatic steatosis, obesity, type 2 diabetes

## Abstract

**Background:**

Nonalcoholic fatty liver disease (NAFLD) is associated with hepatic as well as systemic insulin resistance even in the absence of type 2 diabetes. The extent and pathways through which hepatic inflammation modulates insulin sensitivity in NAFLD are only partially understood. We explored the contribution of hepatic interleukin (IL)‐1 signalling in a novel conditional knockout mouse model and expand the knowledge on this signalling pathway with regard to its liver‐specific functions.

**Methods:**

A high‐fat, high‐carbohydrate diet (HFD) over 12 weeks was used in male hepatocyte‐specific IL‐1 receptor type 1 (IL‐1R1) knockout mice (*Il1r1* ^Hep−/–^) and wild‐type (WT) littermates.

**Results:**

Both genotypes developed an obese phenotype and accompanying macrovesicular hepatic steatosis. In contrast to WT mice, microvesicular steatosis and ballooning injury was less pronounced in HFD‐fed *Il1r1* ^Hep−/–^ mice, and alanine aminotransferase remained in the normal range. This was paralleled by the suppression of injurious and proinflammatory hepatic c‐Jun N‐terminal kinases and extracellular signal‐regulated kinases signalling, stable peroxisome proliferator activated receptor gamma coactivator‐1alpha and farnesoid X receptor‐alpha expression and preservation of mitochondrial function. Strikingly, despite HFD‐feeding *Il1r1* ^Hep−/–^ mice remained highly insulin sensitive as indicated by lower insulin levels, homeostatic model assessment for insulin resistance, higher glucose tolerance, more stable hepatic insulin signalling cascade, and less adipose tissue inflammation compared to the WT.

**Conclusions:**

The current data highlights that hepatocyte IL‐1R1 contributes to hepatic and extrahepatic insulin resistance. Future liver‐directed therapies in NAFLD could have effects on insulin sensitivity when improving hepatic inflammation and IL‐1R1 signalling.

## Graphical abstract

- Hepatic innate immune receptor controls whole body insulin sensitivity
- Intrahepatic and extrahepatic inflammation in a murine model of NAFLD is dampened following deletion of IL‐1R1 in hepatocytes
- IL‐1R1 controls lipid metabolism in the liver

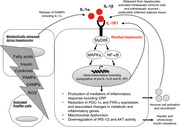

## 1 BACKGROUND

The liver is a central regulator of energy maintenance and contributes to glucose homeostasis. This central metabolic function is achieved through hepatocytes storing and producing glucose depending on the availability of dietary substrates, insulin, and other metabolic hormones. In overnutrition, the levels of circulating fatty acids are increase favouring the development of nonalcoholic fatty liver disease (NAFLD). Furthermore, the aberrant accumulation of triglycerides within the liver is promoted by hepatic insulin resistance, which is characterized by the failure of insulin to suppress glucose production and hepatic de novo fatty acid synthesis. Patients with NAFLD almost universally exhibit systemic insulin and hepatic insulin resistance in addition to adipose tissue and muscle insulin resistance, which are all contributing to the clinical phenotype in diabetes mellitus. Insulin signalling in the liver is critically controlled through activation of protein kinase C‐epsilon (PKC‐ɛ),^1^ c‐Jun N‐terminal kinases (JNK), and also nuclear factor‐kappa B (NF‐κB) in response to lipid metabolites – in particular, diacylglycerol and ceramide ‐, cytokines, microbiota‐derived lipopolysaccharides, and advanced glycation end products.^2^

Extensive evidence supports a role for interleukin (IL)‐1 signalling in the development of insulin resistance and type 2 diabetes.^3^ Both proinflammatory forms of IL‐1 – IL‐1α and IL‐1β – signal through the ubiquitously expressed cell surface IL‐1 receptor type 1(IL‐1R1).^4^ Stimulation of IL‐1R1 leads to the recruitment of an accessory protein (IL‐1RAcP or IL‐1R3), the association of the receptor complex with the adaptor molecule myeloid differentiation primary response 88 (MyD88), and after a series of signalling steps funnelling on the activation of NF‐κB and mitogen‐activated protein kinases (MAPKs) including JNK, extracellular signal‐regulated kinases (ERK), and p38.^5^ Opposing effects are seen with IL‐1 receptor antagonist (IL‐1Ra) that block the binding of IL‐1α or IL‐1β.

Mice harbouring targeted deletions of either IL‐1β, IL‐1R1,^6^, ^7^ activating enzymes of IL‐1β, for example, nucleotide‐binding domain, leucine‐rich‐containing family, pyrin domain‐containing‐3 (NLRP3) inflammasome components,^8^, ^9^, ^10^ or neutrophil serine proteases^11^ are protected from diet‐induced metabolic inflammation and insulin resistance. In a variety of rodent models, prediabetes and obesity were ameliorated by inhibition of IL‐1 signalling or antibodies antagonizing IL‐1β. Both strategies resulted in benefits from a metabolic perspective including improved hyperglycaemia and insulin sensitivity.^12^ Importantly, blockade of IL‐1 signalling in patients with type 2 diabetes with recombinant human IL‐1Ra (Anakinra) has also been shown to improve glycaemic control and β‐cell function and reduced markers of systemic inflammation.^13^ Likewise, monoclonal antibodies antagonizing IL‐1β improve β‐cell secretory function, metabolic control, and inflammation of pancreas and liver.^14^ These data have supported attempts to develop an anti‐IL‐1β vaccine for the treatment of type 2 diabetes.^15^

The importance of hepatic IL‐1 signalling in the regulation of the metabolism is unclear. The current study demonstrates that hepatocyte‐specific loss of the IL‐1R1 does not affect the development of obesity or NAFLD from a high‐fat, high‐carbohydrate diet (HFD) but led to a significant reduction of hepatocyte injury and improved both hepatic and whole‐body insulin sensitivity.

## 2 MATERIAL AND METHODS

### 2.1 Animal model

All animals were held and bred at the animal facility of the University Medical Center Mainz according to the criteria outlined by the ‘Guide for the Care and Use of Laboratory Animals’. Studies were approved by the committee for experimental animal research (Landesuntersuchungsamt Rheinland‐Pfalz, Koblenz, Germany, Approval ID: G‐18‐1‐066). Transgenic *Il1r1* ^Hep−/–^ mice (Alb‐Cre:IL‐1R1^flox/flo^) exhibiting deletion of all signalling‐capable IL‐1R1 isoforms in hepatocytes were generated as described previously.^16^ At 8–10 weeks of age, male *Il1r1* ^Hep−/−^ mice and wild‐type (WT) littermates were fed a high‐fat diet (35.5% w/w crude fat [58 kJ%], metabolizable energy [ME]: 5.45 kcal/g) and drinking water supplemented with fructose (55 % w/v) and glucose (45 % w/v) for 12 weeks.^17^ Gender‐ and age‐matched controls received a matched control diet (CD, 5.4 % w/w crude fat [13 kJ%], ME: 3.74 kcal/g) and plain water. The composition and energy density of the diets (ssniff Spezialdiäten GmbH, Soest, Germany) are listed in Table S1. For the duration of the study, all mice were kept on a 12‐h light/dark cycle with constant temperature (22 ± 2°C) and humidity (55 ± 10%) and with free access to the experimental diets and water. Biometric data including body weight and food consumption was measured weekly. For glucose tolerance testing at week 11 of diet intervention, mice were fasted for 6 h, then a glucose solution (2 mg/kg body weight) was administered by intraperitoneal (i.p.) injection, and blood glucose level was measured at different time points using the Accu‐Chek Aviva system (Roche, Mannheim, Germany). After the 12‐week experimental period, all mice were fasted overnight before sacrificed for collection of blood, liver, and visceral adipose tissue samples. To examine insulin‐stimulated activation of hepatic AKT in vivo, 16–18‐h fasted mice were injected i.p. with 1.5 U/kg body weight insulin (Lilly, Bad Homburg, Germany) and sacrificed at 10‐ or 15‐min following injection for collection of liver tissue. If indicated, recombinant mouse (rm)IL‐1β protein (1 μg, R&D Systems, Minneapolis, MN, USA) was i.p. administered 30 min before the insulin injection.

### 2.2 RNA sequencing and analysis

Total RNA was extracted from liver tissue of naïve male *Il1r1* ^Hep−/–^ mice and WT littermates at 10 weeks of age using the RNeasy Plus Universal Mini Kit (Qiagen, Hilden, Germany). RNA was checked for integrity using Agilent Bioanalyzer and quantified before use. Libraries were prepared using Illumina TruSeq stranded mRNA Library Prep kits and were sequenced on an Illumina NextSeq500 machine in single end mode with 75 bp read length, yielding an average of 40.3 million raw reads per sample. STAR v2.7 with default parameter settings (except –outFilterMismatchNoverLmax 0.04 and –outFilterMismatchNmax 999) was used to align reads to the mouse reference genome GRCm38 (gencode release M25) obtaining a mean unique mapping rate of 77.6%. Read summarization at the gene level was performed using Subread featureCounts v1.6 with default parameter settings. The pairwise differential expression analysis between the *Il1r1* ^Hep−/–^ and WT mice (six replicates each) was performed with the R‐package DESeq2 v.1.26.0. In overall gene expression, the samples showed little differences with respect to their genotype and did not cluster in separated groups in the PCA plot. Thus, analysis was performed in paired sample design, which included the littermate information as a term in the DESeq2 design formula, which accounts for differences between the litters while estimating the effect due to the condition. Genes were tested for evidence that the expression changes between both sample groups was different from zero (i.e., absolute fold change > 1) and were considered significant if obtained *p*‐values were below .05 after adjustment for false discovery rate (fdr).

To qualitatively check for IL‐1R1 gene expression in native liver tissue, we downloaded publicly available single cell RNA‐Seq mRNA baseline datasets of mouse and human from the Single Cell Expression Atlas (https://www.ebi.ac.uk/gxa/sc/home). These datasets include the comprehensive Tabula muris compendium^18^ with 53,759 cells from 20 mouse organs and tissues (https://www.ebi.ac.uk/gxa/sc/experiments/E‐ENAD‐15/results/tsne) as well as two human liver datasets^19^, ^20^ with 17,123 and 10,578 cells, respectively (https://www.ebi.ac.uk/gxa/sc/experiments/E‐MTAB‐10553/results/tsne, https://www.ebi.ac.uk/gxa/sc/experiments/E‐HCAD‐9/results/tsne). The normalized counts matrices were downloaded and merged with the provided experimental meta and annotation data using the R programming language. IL‐1R1 expression counts (Ensembl gene Id ‘ENSMUSG00000026072’ or ‘ENSG00000115594’ for mouse or human, respectively) were summarized by tissue and cell type as annotated by the authors. Cells missing any cell type annotation were summarized as ‘not assigned’ (Tables S2–S4).

### 2.3 Serological analysis, determination of homeostatic model assessment for insulin resistance, and adipose tissue insulin resistance index

Serum was obtained from 16‐ to 18‐h fasted, anesthetized mice by cardiac puncture and assayed for levels of alanine aminotransferase (ALT), triglycerides, total cholesterol, and glucose using standard analyser (Hitachi 917, Roche). ELISA kits were used to measure adiponectin (Thermo Fisher Scientific, Waltham, MA, USA), nonester fatty acids (NEFA, MyBioSource, San Diego, CA, USA), insulin, C‐reactive protein (CRP), and thrombopoietin (TPO, all MilliporeSigma, St. Louis, MI, USA) in serum. The following equations were used to determine homeostatic model assessment for insulin resistance (HOMA‐IR) and adipose tissue insulin resistance index (Adipo‐IR) levels of the mice: HOMA‐IR = [insulin level subsequent to fasting (μIU/ml) × glucose level following fasting (mg/dl)/405] and Adipo‐IR = insulin level subsequent to fasting (pmol/l) × NEFA level following fasting (mmol/l).

### 2.4 Histological analysis of mouse liver tissue and human patient samples

For histological examination of mouse liver tissue representative liver sections were cut, fixed in 4% paraformaldehyde‐PBS, embedded in paraffin, and stained with haematoxylin and eosin (H&E) following standard procedures. Semiquantitative evaluation of steatosis, hepatocellular ballooning, and lobular inflammation in liver tissue was done blinded by an expert hepatopathologist (BKS) by creating the SAF score encompassing an assessment of steatosis (S), activity (A), and fibrosis (F) established by Bedossa et al.^21^ Representative pictures were taken using an Olympus BX45 microscope (Olympus Deutschland, Hamburg, Germany) with a Jenoptik PROGRES GRYPHAX camera (Micro Optimal, Meerbusch, Germany) and the Olympus Image Analysis Software analySIS docu (Olympus Deutschland).

Formalin‐fixed and paraffin‐embedded human liver samples of patients with simple hepatic steatosis (NAFLD activity score [NAS] < 3) and noncirrhotic nonalcoholic steatohepatitis (NASH, NAS > 5) were stained for IL‐1R1 (antibody from Novus Biologicals [Littleton, CO, USA] or Sigma‐Aldrich [St. Louis, MI, USA]) according to the manufacturer's protocols. Controls were stained in the absence of the primary IL‐1R1 antibody. Tissue samples derived from therapeutically indicated liver biopsies were provided by the tissue bank of the University Medical Center Mainz in accordance with the regulations of the tissue biobank and the approval of the ethics committee of University Medical Center Mainz. Written informed consent was obtained from all patients.

### 2.5 Determination of the hepatic triglyceride, protein carbonyl, and malondialdehyde (MDA) content

Hepatic triglycerides were measured using the Triglyceride Quantification Kit (BioVision, Milpitas, CA, USA) according to the manufacturer's instructions. Protein carbonyl and MDA levels in whole liver tissue were detected and quantitated using the OxiSelect™ Carbonyl ELISA Kit (Cell Biolabs, Inc., San Diego, CA, USA) and the Lipid Peroxidation (MDA) Colorimetric Assay Kit (BioVision), respectively, according to the manufacturer's protocols.

### 2.6 Quantitative real‐time‐PCR

Isolation of total RNA, cDNA synthesis and quantitative real‐time polymerase chain reaction (qRT‐PCR) were performed as previously described.^22^ All samples were performed in duplicate. Roche LightCycler software (LightCycler 480 Software Release 1.5.0, Roche) was used to perform advanced relative quantification analysis using the 2^(–ΔΔC(T))^ method. Expression data were normalized to the housekeeping gene glyceraldehyde‐3‐phosphate dehydrogenase (GAPDH, mouse primer sequences from Qiagen, Hilden, Germany, human primer sequences from Eurofins Genomics, Ebersberg, Germany), which was stably expressed and calculated as fold change over expression in WT mice fed with the CD or untreated cells, which was considered 1. The target genes and the primer sequences used for qRT‐PCR (all Eurofins Genomics) are listed in Table S5 and S6.

### 2.7 Protein isolation, immunoblotting, and NF‐κB p65 activity assay

Proteins were isolated and separated as previously described.^22^ Primary antibodies included: actinin, AKT, phospho‐AKT (Ser473), insulin receptor β, IRS‐1, phospho‐IRS‐1 (Ser302) and (Ser307), Lamin B1, LC3B, NF‐κB p65, phospho‐NF‐κB p65 (Ser536), p38 MAPK, phosho‐p38 MAPK (Thr180/Tyr182), p44/42 MAPK (ERK1/2), phospho‐p44/42 MAPK (ERK1/2) (Thr202/Tyr204), SAPK/JNK, phospho‐SAPK/JNK (Thr183/Tyr185), SOCS3 (all obtained from Cell Signaling Technology, Danvers, MA, USA), IL‐1R1 (Sigma‐Aldrich), FXR‐α/NR1H4 (Novus Biologicals, Abingdon, United Kingdom), HO‐1, PGC‐1α, PPAR‐γ (all obtained from Santa Cruz Biotechnology, Dallas, TX, USA), and α‐Tubulin (Abcam, Cambridge, UK). Membranes were exposed to anti‐mouse (DAKO Denmark A/S, Glostrup, Denmark) or anti‐rabbit (Santa Cruz Biotechnology) secondary antibodies conjugated with horseradish peroxidase. PaperPort Professional software v14.0 (Nuance Communications Germany, München, Germany) was used for image acquisition, and the Adobe Acrobat Professional software program (Adobe Systems Incorporated, San Jose, CA, USA) was used to cut immunoblot images to size. No postprocessing of images was performed. Densitometric analysis was performed using National Institutes of Health ImageJ software.

NF‐κB p65 activity was measured in nuclear protein extracts using the TransAM NF‐kappaB Family Kit (Active Motif, Carlsbad, CA, USA) according to the manufacturer's protocol.

### 2.8 Flow cytometric analysis of intrahepatic immune cells

Isolation of intrahepatic immune cells and their flow cytometric analysis was performed as previously described (all antibodies obtained from BioLegend, San Diego, CA, USA).^23^

### 2.9 Quantitative analysis of cytokines and chemokines

C–X–C motif ligand (CXCL)‐1 and CC‐chemokine ligand 2 (CCL2, monocyte chemotactic protein‐1 [MCP‐1]) serum concentrations were measured by ELISA (MilliporeSigma) respectively, Cytometric Bead Array (CBA) assay (BD Biosciences, Franklin Lakes, NJ, USA). IL‐6 was analysed by Mouse IL‐6 ELISA MAX™ (BioLegend) in the supernatant of cultured abdominal adipose tissue (RPMI‐1640 supplemented with 10 % FCS, 2 mM L‐glutamine and 100 U/ml penicillin/streptomycin; all Gibco, Grand Island, NY, USA) after 18 h.

### 2.10 Isolation of primary hepatocytes and in vitro stimulation

Primary hepatocytes were isolated via collagen perfusion and plated in collagen‐coated culture plates as previously described.^22^ Cells were allowed to attach for at least 4 h before switching to fresh, FCS‐free medium and adding all treatments. IL‐1 stimulation was carried out for 3 h or 18 h by adding rmIL‐1α or rmIL‐1β protein (10 ng/ml, both R&D Systems).

To examine whether IL‐1 potentiates free fatty acid toxicity, hepatocytes were exposed to variable concentrations of palmitic acid or oleic acid (both Merck, Darmstadt, Germany) for 24 h in the absence or presence of rmIL‐1α/β (10 ng/ml) in media containing 1% free fatty acid‐free BSA (Merck). For this, each fatty acid was prepared as 100 mM stock solution dissolved in methanol, vortexed, heated at 65°C, and then allowed to complex over night at 37°C with 2.5 mM fatty acid‐free BSA prior adding to culture.^24^ Cell survival following treatment was measured using MTT assay (Merck).

To detect the effects of IL‐1 on insulin signalling, cells were left untreated or treated for 3 h with insulin (100 nM, Merck) in the absence or presence of rmIL‐1α/β (10 ng/ml). Alternatively, cells were prestimulated with rmIL‐1α/β (10 ng/ml) for 18 h followed by a thorough wash with warm PBS and then incubated with insulin (100 nM) for further 3 h. Cell lysates were used as input for qRT‐PCR.

Human HepG2 cells (CLS GmbH, Eppelheim, Germany) and plateable, cryopreserved human primary hepatoctyes (ThermoFisher Scientific, Waltham, MA USA) were cultivated according to the protocols supplied with the cells and stimulated with 100 ng/ml recombinant human (rh)IL‐1α/β protein (R&D Systems). To mimic steatotic conditions, cells were pretreated with a nontoxic dose of BSA‐Oleate (200 μM, MilliporeSigma) for 18 h.

To examine the involvement of specific signalling pathways in mediating IL‐1‐induced effects in murine and human hepatocyte cultures, the NF‐κB inhibitor BAY‐11‐7082 (10 μM), the JNK inhibitor SP600125 (100 μM) or the ERK inhibitor UO126 (50 μM, all obtained from Merck) was added to cell cultures 1 h prior to IL‐1 stimulation.

### 2.11 Statistical analysis

All statistical analyses were performed using GraphPad Prism 7 software (GraphPad Software, La Jolla, CA, USA). All results were initially submitted to Shapiro‐Wilk normality test for normality and to Levene's test for homogeneity of variance. Comparisons between two experimental groups were carried out using the unpaired and two‐tailed Student's *t* test to determine statistical significance of differences. Multiple groups were analysed by two‐way analysis of variance (ANOVA), followed by Bonferroni multiple comparison post hoc tests or Kruskal‐Wallis H test followed by Mann‐Whitney U test with a Bonferroni correction when the assumptions for ANOVA were not met. The results with a *p* value of < .05 were considered to be significant. All data are shown as mean ± standard error of mean (SEM) to determine the precision and differences of means and statistically significant values were assumed with *^/$^ *p* < .05, **^/$$^ *p* < .01, ***^/$$$^ *p* < .001.

## 3 RESULTS

### 3.1 Expression of IL‐1R1 in a murine high‐fat, high‐carbohydrate diet model

Various preclinical studies in rodents and humans support a role of IL‐1 signalling in obesity‐related NAFLD. In a murine model of NAFLD, employing an obesogenic HFD over 12 weeks we observed a 3.6 (± 0.3)‐fold upregulation of IL‐1R1 mRNA in liver tissue (*p* < .01 for HFD vs. CD using unpaired, two‐tailed Student's *t* test, *n* = 7 mice/group, data not shown). However, the specific role of the IL‐1R1 pathway in hepatocytes on metabolic derangement is yet to be elucidated.

Therefore, we aimed to study the development of obesity and accompanying NAFLD in hepatocyte‐specific IL‐1R1 knockout mice (*Il1r1* ^Hep−/–^) and their WT littermates.^16^ In contrast to global IL‐1R1 knockout mice that exhibit mature‐onset obesity, insulin resistance, and glucose intolerance,^25^ naïve *Il1r1* ^Hep−/–^ mice did not exhibit a metabolic or liver phenotype up until 6 months (Figure S1A–C).^16^ In addition, RNA‐seq analysis of hepatic tissue derived from naïve *Il1r1* ^Hep−/–^ and WT mice for further characterization of the transgenic model identified a total of 10 significantly differentially expressed genes from comparison of *Il1r1* ^Hep−/–^ versus WT mice, with seven down‐ and three up‐regulated genes in the *Il1r1* ^Hep−/–^ group (Figure S2A and Table S7). Thus, targeted knockout of *Il1r1* in hepatocytes alters hepatic constitutive expression of only a few other genes with *Socs3* (suppressor of cytokine signalling [SOCS] 3) being the most upregulated one in *Il1r1* ^Hep−/–^ livers, which we also validated on protein level (Figure S2B).

**FIGURE 1:**
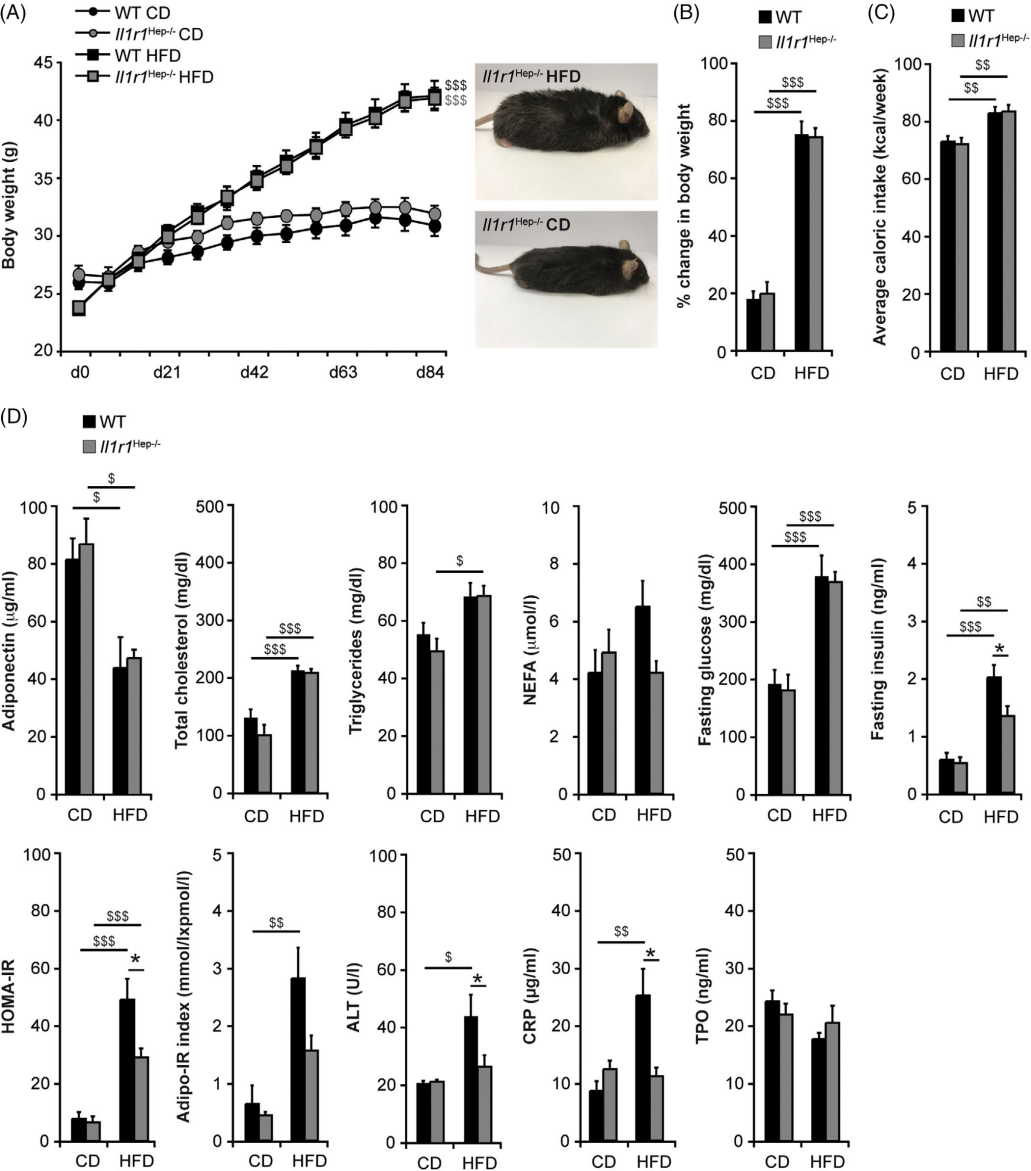
Body weight gain and systemic metabolic alterations in *Il1r1* ^Hep−/–^ and WT mice fed a high‐fat, high‐carbohydrate diet for 12 weeks. Eight to 10‐week‐old, male *Il1r1* ^Hep−/–^ mice and WT littermates were metabolically challenged with a high‐fat, high‐carbohydrate diet (HFD) or received a corresponding control diet (CD) for 12 weeks. (A) Body weight curve, (B) % change in body weight, and (C) mean caloric intake during the entire experimental period. (D) Serum samples were drawn from fasted mice to measure the levels of adiponectin, total cholesterol, triglycerides, NEFA, glucose, insulin, ALT, CRP, and TPO after 12 weeks of dietary feeding and to determine HOMA‐IR and Adipo‐IR indices. Data in (A–C) represent mean of *n* = 7 *Il1r1* ^Hep−/–^ CD, *n* = 10 WT CD, *n* = 15 *Il1r1* ^Hep−/–^ HFD, and *n* = 15 WT HFD mice ± SEM. Data in (D) represent mean of *n* = 4 *Il1r1* ^Hep−/–^ CD, *n* = 3 WT CD, *n* = 7 *Il1r1* ^Hep−/–^ HFD and *n* = 6 WT HFD mice ± SEM (adiponectin), *n* = 6 *Il1r1* ^Hep−/–^ CD, *n* = 8 WT CD, *n* = 11 *Il1r1* ^Hep−/–^ HFD and *n* = 11 WT HFD mice ± SEM (total cholesterol, triglycerides), *n* = 5 *Il1r1* ^Hep−/–^ CD, *n* = 5 WT CD, *n* = 6 *Il1r1* ^Hep−/–^ HFD and *n* = 9 WT HFD mice ± SEM (NEFA, Adipo‐IR), *n* = 7 *Il1r1* ^Hep−/–^ CD, *n* = 10 WT CD, *n* = 15 *Il1r1* ^Hep−/–^ HFD and *n* = 15 WT HFD mice ± SEM (glucose, insulin, HOMA‐IR, and ALT), resp. *n* = 4 *Il1r1* ^Hep−/–^ CD, *n* = 6 WT CD, *n* = 11 *Il1r1* ^Hep−/–^ HFD and *n* = 9 WT HFD mice ± SEM (CRP, TPO). *p < .05 for *Il1r1* ^Hep−/–^ versus WT and ^$^ *p* < .05, ^$$^ *p* < .01, ^$$$^ *p* < .001 for CD versus HFD using two‐way method of ANOVA (A–D) and Kruskal‐Wallis H test (D [Insulin, HOMA‐IR, ALT, and TPO]) following the Bonferroni multiple comparison tests and Mann‐Whitney tests with a Bonferroni correction, respectively.

Following 12 weeks of HFD‐feeding *Il1r1* ^Hep−/–^ mice and their WT littermates exhibited a significant weight gain compared to CD, with comparable body weight gain in the two genotypes (Figure 1A,B). In the HFD groups, the daily caloric intake was significantly higher compared to CD, but no differences were observed between the genotypes (Figure 1C). The development of HFD‐induced obesity was paralleled by a significant decrease in serum adiponectin levels and elevations in serum total cholesterol, triglycerides, and glucose in overnight‐fasted mice with no differences between *Il1r1* ^Hep−/–^ and WT mice (Figure 1D). Despite hyperglycaemia, fasting insulin levels were significantly lower in HFD‐fed *Il1r1* ^Hep−/–^ mice compared to WT littermates (Figure 1D). This translated into retained insulin sensitivity as measured by a significantly lower average homeostatic model assessment for insulin resistance (HOMA‐IR) score in *Il1r1* ^Hep−/–^ mice (Figure 1D). Likewise, the increase in adipose tissue insulin resistance index (Adipo‐IR) was more pronounced in the WT compared to the *Il1r1* ^Hep‐/^ mice, albeit circulating nonester fatty acid (NEFA) levels were not significantly affected by diet or genotype (Figure 1D).

Accompanying these metabolic derangements, WT mice on HFD showed mildly elevated alanine aminotransferase (ALT) while being in the range of normal in *Il1r1* ^Hep−/–^ mice (Figure 1D). An inflammatory phenotype in HFD‐fed WT mice was also reflected by moderately elevated C‐reactive protein (CRP) levels (Figure 1D). In addition, WT mice on HFD exhibited a reduction of thrombopoietin (TPO) compared to HFD‐fed *Il1r1* ^Hep−/–^ mice and CD‐fed controls (Figure 1D), suggesting more circulating platelets degrading TPO.

### 3.2 Regulation of hepatic steatosis and lipotoxicity through IL‐1R1 in NAFLD

**FIGURE 2:**
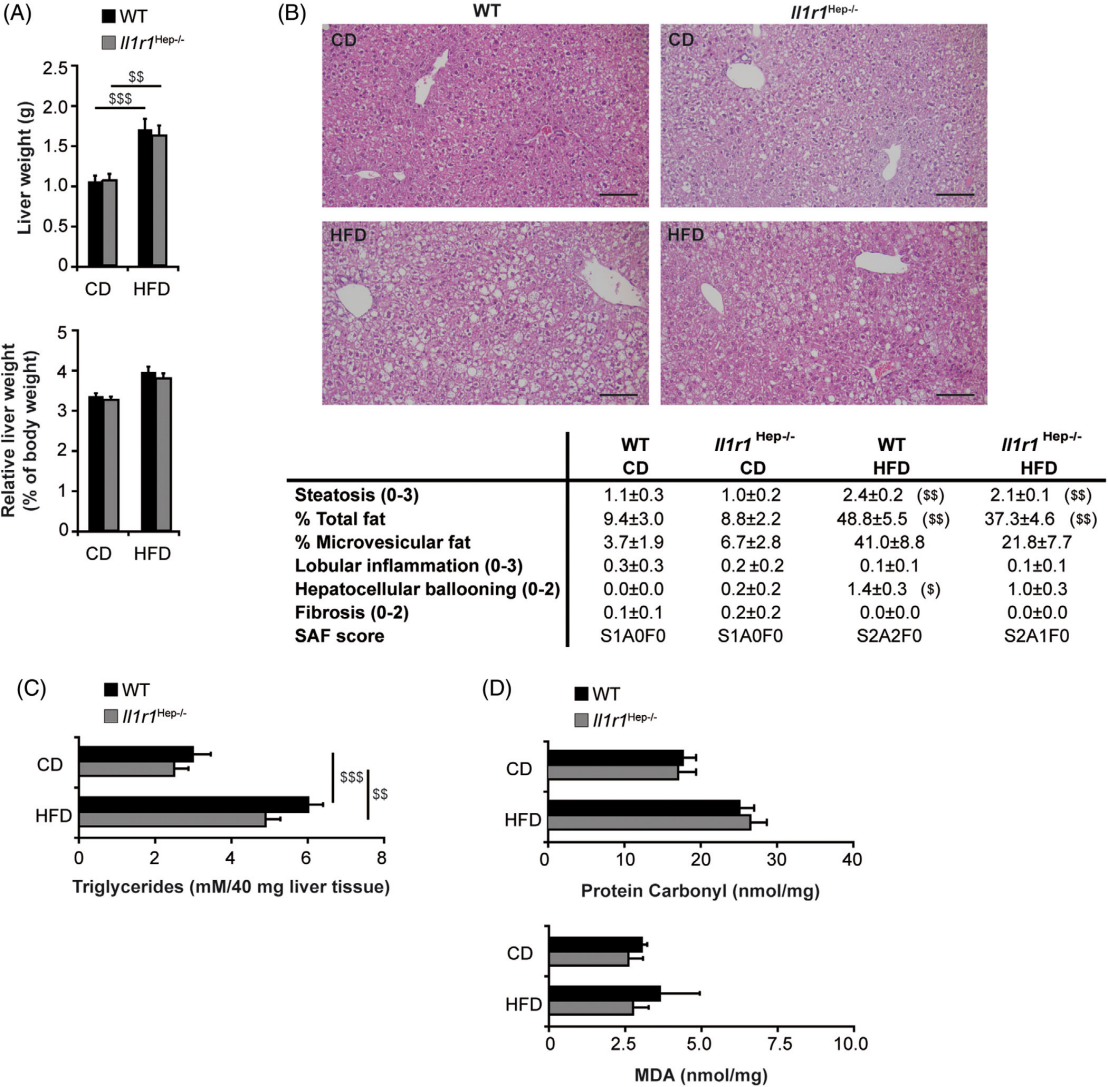
Effects of HFD feeding on the development of NAFL in *Il1r1* ^Hep−/–^ and WT mice. (A) Absolute liver weight, liver‐to‐body‐weight ratio, (B) representative liver histology by H&E staining (scale bar: 100 μM) and SAF score, (C) biochemically determined liver triglyceride and (D) protein carbonyl and MDA content of *Il1r1* ^Hep−/–^ and WT mice fed with the diets for 12 weeks. Data in A represent mean of *n* = 7 *Il1r1* ^Hep−/–^ CD, *n* = 10 WT CD, *n* = 15 *Il1r1* ^Hep−/–^ HFD and *n* = 15 WT HFD mice ± SEM, in B and C *n* = 6 *Il1r1* ^Hep−/–^ CD, *n* = 8 WT CD, *n* = 11 *Il1r1* ^Hep−/–^ HFD and *n* = 11 WT HFD mice ± SEM, and in D *n* = 4 *Il1r1* ^Hep−/–^ CD, *n* = 4 WT CD, *n* = 8 *Il1r1* ^Hep−/–^ HFD and *n* = 8 WT HFD mice ± SEM. ^$^ *p* < .05, ^$$^ *p* < .01, ^$$$^ *p* < .001 for CD versus HFD using two‐way method of ANOVA (A and C) and Kruskal‐Wallis H test (B and D) following the Bonferroni multiple comparison tests and Mann Whitney tests with a Bonferroni correction, respectively. There was no statistical difference for *Il1r1* ^Hep−/–^ versus WT with respect to these parameters

All mice regardless of genotype developed hepatomegaly at 12 weeks from HFD feeding (Figure 2A). Histological analysis indicated mixed macro‐ and microvesicular steatosis (Figure 2B). While macrovesicular steatosis was comparable between the genotypes, *Il1r1* ^Hep−/–^ mice demonstrated reduced amounts of microvesicular‐type fat on blinded analysis. Likewise, hepatocyte ballooning was less prominent in transgenic livers. Importantly, despite being steatotic and ballooning degeneration, no inflammation or fibrosis suggestive of inflammatory nonalcoholic steatohepatitis (NASH) was observed histologically in *Il1r1* ^Hep−/–^ nor WT livers after 12‐week‐HFD feeding, consistent with this being a relative mild model of obesogenic NAFLD.^26^ This was supported by grading and staging of liver histology using the steatosis, activity, and fibrosis (SAF) score (Figure 2B). Quantification of hepatic triglyceride content in the HFD‐fed showed a two‐ to threefold increase compared to CD‐fed animals. When comparing HFD‐fed mice, there was no significant difference between the genotypes (Figure 2C). Overall, protein carbonyls and malondialdehyde (MDA) – indicative of tissue oxidative stress – were slightly increased from HFD feeding without significant differences between the genotypes (Figure 2D). Hence, despite comparable oxidative stress, we found less pronounced metabolic and injurious alterations in *Il1r1* ^Hep−/–^ mice on HFD.

The findings on hepatocellular lipotoxicity were replicated ex vivo. Treatment of primary WT hepatocytes with palmitic acid led to a significant reduction of cell viability on MTT assay when adding rmIL‐1α/β protein (Figure S3A). By contrast, rmIL‐1α/β did not increase the susceptibility to oleic acid, which was generally less toxic (Figure S3B). These results were similar to those in human HepG2 cells (data not shown). Thus, IL‐1 signals may exacerbate the cell death response elicited by toxic fatty acids among hepatocytes by affecting injurious intracellular signalling pathways.

### 3.3 Regulation of lipid metabolism and mitochondrial function through hepatic IL‐1R1

According to our in vitro studies in human HepG2 cells and primary human hepatocytes, IL‐1 signals can favour hepatic lipid deposition, among others, by upregulation of sterol regulatory element binding protein‐1c (SREBP‐1c) – the key regulator of de novo lipogenesis – in parallel to downregulation of mitochondrial fatty acid β‐oxidation‐associated genes such as peroxisome proliferator‐activated receptor‐alpha (PPAR‐α) and carnitine palmitoyltransferase 1 (CPT1) (Figure S4A–C). However, gene regulation might be also affected by additional metabolic signals, as IL‐1‐induced CPT1 downregulation was abolished in BSA–oleate induced steatotic hepatocytes (Figure S4A,C).

**FIGURE 3:**
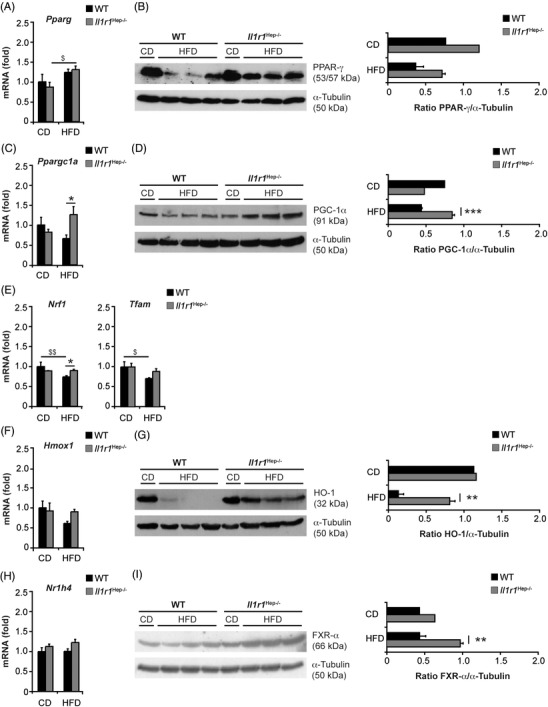
HFD‐induced changes in hepatic lipid metabolism and mitochondrial function of *Il1r1* ^Hep−/–^ and WT mice. Expression analysis of PPAR‐γ (A and B), PGC‐1α (C and D), NRF‐1, and TFAM (E), HO‐1 (F and G), and FXR‐α (H and I) in liver whole tissue lysates from *Il1r1* ^Hep−/–^ and WT mice following 12‐week feeding of the HFD or the CD by qRT‐PCR (A, C, E, F, and H) and immunoblotting (B, D, G, and I). Data in A, C, E, F, and H represent mean of *n* = 4 *Il1r1* ^Hep−/–^ CD, *n* = 4 WT CD, *n* = 7 *Il1r1* ^Hep−/–^ HFD, and *n* = 7 WT HFD mice ± SEM. In B, D, G, and I representative immunoblots with densitometric analysis are shown. *p < .05, **p < .01, ***p < .001 for *Il1r1* ^Hep−/–^ versus WT and ^$^ *p* < .05 for CD versus HFD using two‐way method of ANOVA following the Bonferroni multiple comparison tests (A, C, E, F, and H) and unpaired, two‐tailed Student's *t*‐test (B, D, G, and I)

Therefore, next, we investigated the expression of genes involved in lipid metabolism in vivo. Among the studied genes encoding key transcriptional regulators and enzymes involved in fatty acid uptake (fatty acid translocase [FAT/CD36], encoded by *Cd36*), de novo lipogenesis (SREBP‐1c, carbohydrate‐responsive element‐binding protein [ChREBP], acetyl‐CoA carboxylase [ACC], fatty acid synthase [FAS], and stearoyl‐CoA desaturase 1 [SCD1], encoded by *Sreb1f*, *Mlixpl*, *Acaca*, *Fasn*, and *Scd1*, respectively) – mitochondrial fatty acid β‐oxidation (PPAR‐α and CPT1, encoded by *Ppara* and *Cpt1a*, respectively) and export (microsomal triglyceride transfer protein [MTP], encoded by *Mttp*) – no significant differences in the hepatic expression levels were observed when comparing the two genotypes (Figure S5). Likewise, steatogenic PPAR‐γ (encoded by *Pparg*) was comparable at the level of mRNA (Figure 3A). On the contrary, the amount of PPAR‐γ protein was decreased following HFD feeding to a lower extent in *Il1r1* ^Hep−/–^ mice (n.s.; *p = .06 for WT HFD vs. *Il1r1* ^Hep−/–^ HFD using unpaired, two‐tailed Student's *t* test; Figure 3B). In addition to its role in lipid homeostasis, the loss of PPAR‐γ protein in hepatocytes has been associated with a deregulation of mitochondrial metabolism and an aggravation of insulin resistance.^27^ Indeed, we detected that also PPAR‐γ coactivator‐1alpha (PGC‐1α encoded by *Ppargc1a*) – a critical transcriptional co‐activator of PPAR‐γ and many additional transcription factors assuring mitochondrial integrity/function and metabolic homeostasis – was strongly reduced in WT livers in response to the HFD both at the mRNA and protein level. In contrast, hepatic PGC‐1α mRNA levels were increased in *Il1r1* ^Hep−/–^ mice and this translated into significantly higher protein expression of PGC‐1α in HFD‐fed *Il1r1* ^Hep−/–^ mice (Figure 3C,D). In parallel, we observed that the HFD‐induced suppression of the PGC‐1α downstream transcription factor nuclear respiratory factor‐1 (NRF‐1, encoded by *Nrf1*) and its target gene mitochondrial transcription factor A (TFAM, encoded by *Tfam*) – also key regulatory factors involved in mitochondrial metabolism and organelle biogenesis – was less pronounced in *Il1r1* ^Hep−/–^ mice compared to the WT (Figure 3E). Improved mitochondrial integrity seemed to be also reflected by heme oxygenase (HO)‐1, a further downstream target of PGC‐1α, which plays a protective role against the oxidant injury accompanying inflammatory processes in NAFLD^28^ and suppresses steatosis by modifying fatty acid turnover, among others, via Sirtuin 1.^29^ Hepatic gene and protein expression of HO‐1 (encoded by *Hmox1*) were significantly reduced in WT mice fed wi the HFD compared to *Il1r1* ^Hep−/–^ littermates, in which HO‐1 expression levels were hardly affected by the HFD (Figure 3F,G). In parallel, the gene expression levels of Sirtuin 1 (encoded by *Sirt1*) tended to decline in the WT HFD group and to increase in the *Il1r1* ^Hep−/–^ HFD group (Figure S6A). Interestingly, given that Sirtuin 1 favours the clearance of excessive lipid droplets and damaged cell components via autophagy,^30^ we detected that *Il1r1* ^Hep−/–^ mice consistently showed an induction of the autophagy‐related microtubule‐associated protein 1 light chain 3 (MAPLC3/LC3) B‐II^31^ in response to the HFD in contrast to WT littermates (Figure S6B). From this data, it appears that IL‐1/IL‐1R1 signalling in hepatocytes enhances mitochondrial burden under metabolic stress.

Additionally, we observed that the hepatic mRNA and protein levels of the nuclear bile acid receptor farnesoid X receptor‐alpha (FXR‐α, encoded by *Nr1h4*) that is under transcriptional control of PGC‐1α^32^ was significantly higher in *Il1r1* ^Hep−/–^ mice compared to WT littermates after HFD‐feeding (Figure 3H,I). These transcriptional changes were also detectable in the FXR‐α target gene *Ces1d* – encoding the triglyceride hydrolase carboxylesterase 1 (CES1) (Figure S5). This suggests that stable FXR‐α expression in *Il1r1* ^Hep−/–^ mice might reduce the levels of hepatic triglycerides, among others through modulation of hepatic hydrolysis in a CES1‐dependent manner,^33^ in addition to regulation of mitochondrial function and autophagy. However, other hydrolysis‐related genes such as *Apoc2* and *Lpl* (encoding apolipoprotein C2 [APOC2] and lipoprotein lipase [LPL], respectively) remained unaffected by genotype (Figure S5).

The findings on a IL‐1‐PGC‐1α/FXR‐α axis replicated in vitro in primary WT hepatocytes in response to rmIL‐1α or rmIL‐1β protein with a significant inhibition of PGC‐1α and FXR‐α (Figure S7A,B). Likewise, HepG2 cells and primary human hepatocytes showed a rapid IL‐1‐induced downregulation of PGC‐1α and FXR‐α (Figure S8A,B) – also under steatotic conditions (Figure S8C). On contrary, the repression of the PGC‐1α and FXR‐α genes was totally, respectively, in part prevented in the presence of the chemical ERK1/2 inhibitor UO126 (Figure S8A), pointing to a role of IL‐1R1/ERK signalling in regulating PGC‐1α and FXR‐α levels under inflammatory conditions.

### 3.4 Role of hepatocellular IL‐1R1 on hepatic insulin resistance and glucose homeostasis

Hepatic steatosis is strongly associated with impaired glucose tolerance and insulin resistance, whereby particular defects in mitochondrial function have been shown to correlate with hepatic insulin resistance. Moreover, in addition to inflammatory factors, hepatic SOCS3 has been implicated with insulin signalling depending on the metabolic and inflammatory state.^34^ The suppression of SOCS3 following HFD feeding was more pronounced in WT compared to *Il1r1* ^Hep−/–^ mice (Figure S2B).

**FIGURE 4:**
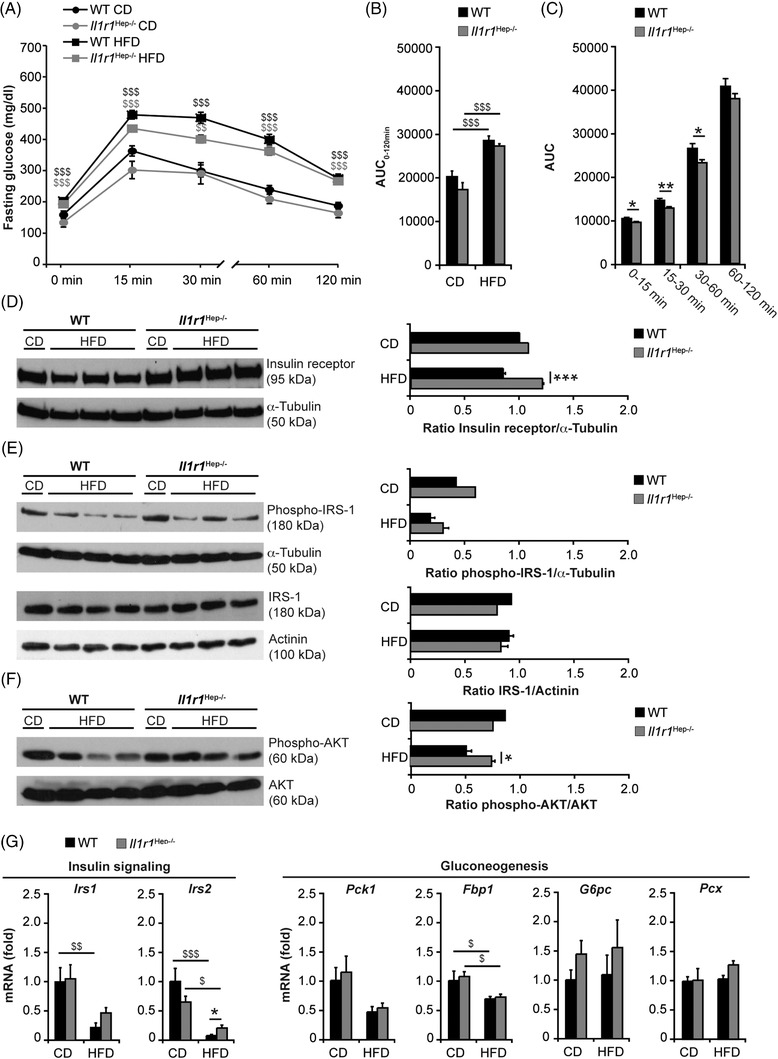
Insulin signalling and glucose metabolism in HFD‐fed *Il1r1* ^Hep−/–^ and WT mice after 12 weeks. (A) I.p. glucose tolerance testing in *Il1r1* ^Hep−/–^ and WT mice following 11‐week feeding of the HFD or the CD, (B) the area under the curve (AUC, 0–120 min), calculated using the trapezoidal rule, in all experimental groups, and (C) AUC of first (0–15 min), second (15–30 min), third (30–60 min), and fourth phase (60–120 min) in HFD‐fed *Il1r1* ^Hep−/–^ mice vs. WT littermates. Molecules involved in hepatic insulin signalling were assessed by immunoblotting of (D) insulin receptor, (E) phospho‐IRS‐1 (Ser307) and total IRS‐1, (F) phospho‐AKT (Ser473) and total AKT following insulin injection, and (G) qRT‐PCR analysis of genes encoding IRS‐1, IRS‐2, and the gluconeogenic enzymes PEPCK, FBP1, G6PC, and PC in liver whole tissue lysates prepared from mice fed with the HFD or the CD for 12 weeks. Data in A–C represent mean of *n* = 6 *Il1r1* ^Hep−/–^ CD, *n* = 8 WT CD, *n* = 11 *Il1r1* ^Hep−/–^ HFD, and *n* = 11 WT HFD mice ± SEM. In D–F representative immunoblots with densitometric analysis are shown. Data in G represent mean of *n* = 4 *Il1r1* ^Hep−/–^ CD, *n* = 4 WT CD, *n* = 7 *Il1r1* ^Hep−/–^ HFD and *n* = 7 WT HFD mice ± SEM. *p < .05, **p < .01, ***p < .001 for *Il1r1* ^Hep−/–^ versus WT and ^$^ *p* < 0.05, ^$$^ *p* < 0.01, ^$$$^ *p* < .001 for CD versus HFD using two‐way method of ANOVA following the Bonferroni multiple comparison tests (B and G) and unpaired, two‐tailed Student's *t*‐test (C–F)

Supporting the finding of retained insulin sensitivity in *Il1r1* ^Hep−/–^ mice despite HFD feeding (Figure 1D), blood glucose was more responsive to insulin injection (*Il1r1* ^Hep−/–^ HFD vs. WT HFD: −21.9% [± 3.1 %] vs. −8.0% [± 12.0%] reduction of blood glucose levels at 15 min after i.p. insulin injection, *n* = 4 mice/group, data not shown) and glucose tolerance – assessed by i.p. glucose tolerance test – was improved in *Il1r1* ^Hep−/–^ mice compared to WT littermates fed with the HFD (Figure 4A–C). Next, key regulators of hepatic insulin signalling were assessed. Liver expression of the insulin receptor in transgenic mice fed with the HFD was significantly higher compared to the WT (Figure 4D). HFD‐induced reduction in Ser307‐phosphorylation of insulin receptor substrate (IRS)‐1 and total IRS‐1 protein expression (Figure 4E) were comparable in both genotypes, while both IRS‐1 and IRS‐2 mRNA levels decreased from HFD‐feeding, but to a lesser extent in the transgenic mice compared to the WT mice (Figure 4G). In parallel, insulin‐induced Ser473‐phosphorylation of the downstream effector AKT was significantly more pronounced in the liver tissue of HFD‐fed *Il1r1* ^Hep−/–^ mice, while total AKT was unaffected by diet and genotype (Figure 4F). Exploration of gluconeogenic enzymes by qRT‐PCR revealed that *Pck1* – encoding phosphoenolpyruvate carboxykinase (PEPCK), the rate‐limiting step in hepatic gluconeogenesis – and *Fbp1* – encoding fructose‐1,6‐bisphosphatase (FBP1) – decreased comparably in both genotypes in response to the HFD. In contrast, the glucose‐6‐phosphatase (G6Pase) and pyruvate carboxylase (PC)‐encoding genes *G6pc* and *Pcx* were unaffected by diet or genotype (Figure 4G).

The reduced insulin responsiveness from HFD was also reflected by the downregulation of IRS‐1 and IRS‐2 mRNA at the level of visceral adipose tissue, that tended to be more pronounced in the WT in comparison to *Il1r1* ^Hep−/–^ mice (Figure S9), in parallel to a trend towards higher expression levels of the glucose transporter 4 (GLUT4, encoded by *Slc2a4*) in the adipose tissues of *Il1r1* ^Hep−/–^ mice than in those of WT littermates fed with the HFD (Figure S9). These findings indicate that IL‐1R1 deletion in hepatocytes maintains physiological insulin sensitivity systemically and at the level of the liver during a 12‐week HFD‐feeding model.

### 3.5 Regulation of MAPK and NF‐κB signalling through IL‐1R1 in the hepatic compartment

**FIGURE 5:**
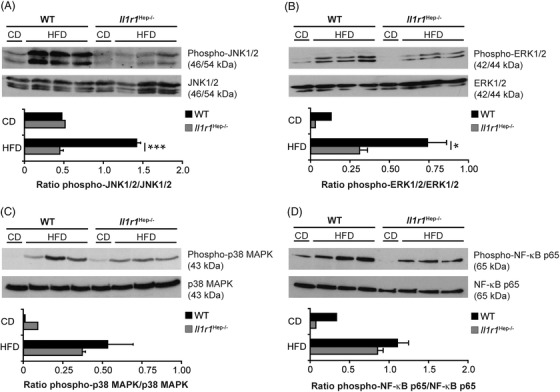
HFD‐induced alterations of MAPK and NF‐κB p65 signalling in the liver of *Il1r1* ^Hep−/–^ and WT mice. Immunoblotting of (A) phospho‐JNK1/2 (Thr183/Tyr185), (B) phospho‐ERK1/2 (Thr202/Tyr204), (C) phosho‐p38 MAPK (Thr180/Tyr182), (D) phospho‐NF‐κB p65 (Ser536) and total (A) JNK1/2, (B) ERK1/2, (C) p38 MAPK, and (D) NF‐κB p65 protein in liver whole tissue lysates of the different experimental groups after 12 weeks of dietary feeding. In A‐D representative immunoblots with densitometric analysis are shown. *p < .05, ***p < .001 for *Il1r1* ^Hep−/–^ versus WT using unpaired, two‐tailed Student's *t*‐test (A and B). There was no statistically significant difference between *Il1r1* ^Hep−/–^ and WT mice on HFD with respect of the parameters in C and D

The MAP kinase superfamily regulates metabolic inflammation and cell injury during the development of insulin resistance in the liver.^35^, ^36^, ^37^ Immunoblotting revealed that the activation/phosphorylation of JNK1/2, ERK1/2, and p38 MAPK in liver tissue was substantially increased in both genotypes from the HFD as compared with CD‐fed controls but was more pronounced in WT mice (Figure 5A–C). Primarily phosphorylation of JNK1/2 and ERK1/2 was significantly suppressed in *Il1r1* ^Hep−/–^ mice. Total JNK1/2, ERK1/2, and p38 MAPK protein were unaltered from diet and genotype (Figure 5A–C). In addition, HFD feeding activated hepatic NF‐κB p65, as measured by Ser536‐phosphorylation, whereby this also occurred to a lesser extent in the hepatic tissue of HFD‐fed *Il1r1* ^Hep−/–^ mice compared to WT littermates but without reaching statistical significance (Figure 5D). These data showed that deletion of IL‐1R1 from hepatocytes reduces HFD‐induced JNK and ERK signalling in the NAFL and in this way might slow the development of hepatic steatosis, concomitant hepatocellular injury and insulin resistance.

However, despite aberrant activation of inflammatory signalling pathways from HFD feeding, there was no development of a severe NASH‐like phenotype. In line with the histological data (Figure 2B), qRT‐PCR‐based gene expression analyses of inflammatory and chemotactic cytokines (including tumour necrosis factor [TNF]‐α, IL‐6, IL‐1α, IL‐1β, IL‐1Ra, CCL2, CXCL‐1, and CXCL‐2 – encoded by *Tnfa*, *Il6*, *Il1a*, *Il1b*, *Il1rn*, *Ccl2*, *Cxcl1*, and *Cxcl2*) and FACS‐based quantification of intrahepatic immune cell subsets including CD45^+^F4/80^+^ macrophages (Figure S10A,B) showed that most of the inflammatory markers were not or only mildly elevated due to 12‐week‐HFD feeding. However, there was a noticeable induction of CXCL‐1/2 at both mRNA and protein levels in liver tissue respectively, serum of HFD‐fed WT mice, which was less pronounced in *Il1r1* ^Hep−/–^ mice (Figure S10A,C). Supporting this finding, IL‐1α/β protein significantly increased CXCL1 and CXCL2 mRNA levels in isolated primary WT hepatocytes in vitro (Figure S7D,E) as well as their functional homolog CXCL8 in human HepG2 cells and primary hepatocytes in an NF‐κB‐dependent manner (Figure S8D,E), pointing to a mild immunomodulatory activity of the hepatocyte IL‐1R1 pathway in early stage NAFLD. Likewise, hepatic gene expression levels of the macrophage mannose receptor CD206 (encoded by *Mrc1*) and Arginase‐1 (ARG1, encoded by *Arg1*) were higher in HFD‐fed *Il1r1* ^Hep−/–^ mice than in the WT, suggestive of a polarization of intrahepatic macrophages to an M2 phenotype in *Il1r1* ^Hep−/–^ mice (Figure S10D).

### 3.6 Evidence of adipose tissue inflammation modulated through hepatic IL‐1R1 signalling

**FIGURE 6:**
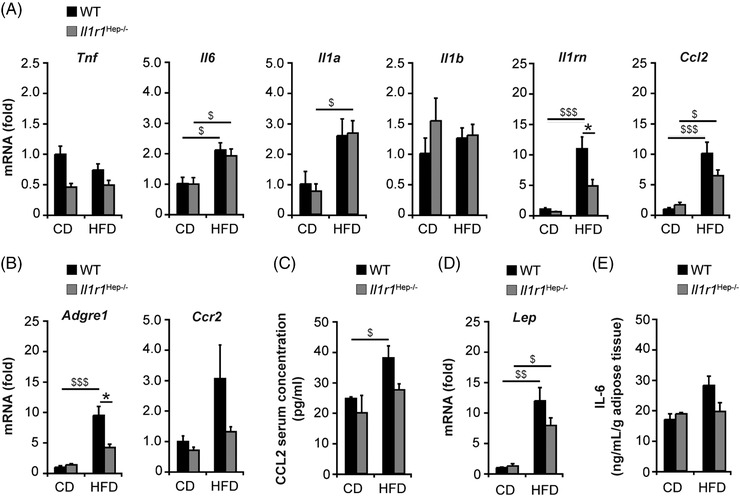
Insulin resistance is accompanied by adipose tissue inflammation in HFD‐fed mice. Inflammation including macrophage recruitment into the obese adipose tissue was assessed by qRT‐PCR‐based gene expression analyses of (A) cytokines, chemokines, (B) the macrophage‐marker F4/80, CCR2, (C) serum levels of CCL2, (D) relative hepatic mRNA expression of leptin, and (E) release of IL‐6 from ex vivo‐cultured adipocytes. Data in A, B, D represent mean of *n* = 5 *Il1r1* ^Hep−/–^ CD, *n* = 4 WT CD, *n* = 8 *Il1r1* ^Hep−/–^ HFD and *n* = 7 WT HFD mice ± SEM, in C *n* = 4 *Il1r1* ^Hep−/–^ CD, *n* = 4 WT CD, *n* = 6 *Il1r1* ^Hep−/–^ HFD and *n* = 6 WT HFD mice ± SEM, and in E mean of duplicate cultures of *n* = 3 *Il1r1* ^Hep−/–^ CD, *n* = 3 WT CD, *n* = 8 *Il1r1* ^Hep−/–^ HFD and *n* = 4 WT HFD mice ± SEM. *p < .05 for *Il1r1* ^Hep−/–^ versus WT and ^$^ *p* < .05, ^$$^ *p* < .01, ^$$$^ *p* < .001 for CD versus HFD using two‐way method of ANOVA following the Bonferroni multiple comparison tests (A–E)

Adipose tissue inflammation contributes to the clinical phenotype in obesity‐associated insulin resistance.^38^ While we did not observe significant hepatic necroinflammation (Figure 2B and Figure S10), the adipose tissue from HFD‐fed mice expressed markedly higher levels of IL‐6, IL‐1α, IL‐1Ra, and CCL2 compared to the CD groups, while TNF‐α and IL‐1β mRNA expression levels appear to remain unaffected by diet and genotype (Figure 6A). Remarkably, the upregulation of IL‐1Ra and CCL2 was less pronounced in *Il1r1* ^Hep−/–^ mice. Likewise, the induction of *Adgre1* – encoding the macrophage‐marker F4/80 – in adipose tissue of HFD‐fed *Il1r1* ^Hep−/–^ mice was significantly lower than in the WT HFD group (Figure 6B). Albeit not reaching statistical significance, this also appeared to apply to the expression of C‐C chemokine receptor type 2 (CCR2), which mediates monocyte/macrophage chemotaxis to the expanding adipose tissue (Figure 6B). In line with this, we found higher circulating CCL2 levels in the WT than *Il1r1* ^Hep−/–^ mice fed with the HFD (Figure 6C). Additionally, the adipocyte‐derived hormone leptin (encoded by *Lep*), which induces the activation of recruited and adipose tissue‐resident immune cells, was significantly upregulated in adipose tissue from HFD‐feeding compared to CD‐feeding, 12.0‐fold ± 2.1 above control in the WT and 6.1‐fold ± 1.0 above control in transgenic mice (Figure 6D), and ex vivo adipose tissue cultures derived from HFD‐fed WT mice exhibited higher IL‐6 levels than those from HFD‐fed *Il1r1* ^Hep−/–^ mice or CD‐fed controls (Figure 6E). Altogether, these data suggest that the deletion of the hepatic IL‐1R1 retains insulin sensitivity involving reduced adipose tissue inflammation.

### 3.7 The IL‐1 signal impairs insulin action in hepatocytes

**FIGURE 7:**
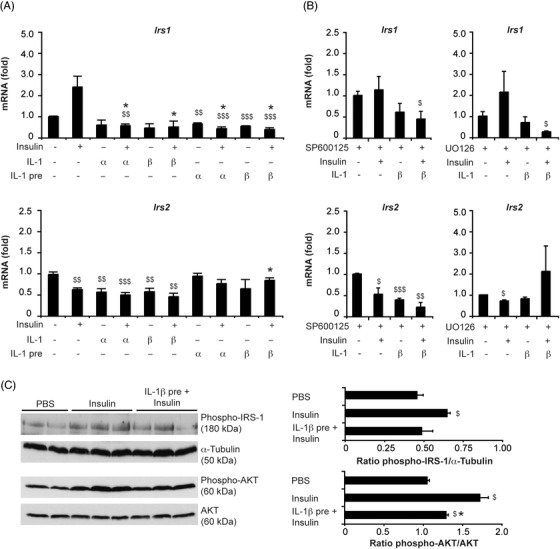
IL‐1 signals alter the expression of IRS‐1 and IRS‐2 in primary hepatocytes and interfere with hepatic insulin signalling in vivo. Relative mRNA expression of IRS‐1 and IRS‐2 in primary WT hepatocytes (A) following ex vivo 3‐h‐stimulation with insulin (100 nM) in the absence or presence of rmIL‐1α/β (10 ng/ml) or after 18‐h‐pretreatment with rmIL‐1α/β (10 ng/ml, IL‐1 pre). (B) To examine the involvement of MAPK pathways in mediating IL‐1‐induced effects, the JNK inhibitor SP600125 (100 μM) or the ERK1/2 inhibitor UO126 (50 μM) was added to hepatocyte cultures 1 h prior to 3‐h‐stimulation with insulin ± rmIL‐1β. (C) Hepatic activation of IRS‐1 (phosphorylation at Ser302) and AKT (phosphorylation at Ser473) was evaluated in 8‐week‐old, male WT mice either treated with vehicle (PBS) or insulin (1.5 U/kg body weight insulin i.p.) at 10 min by Western blot analysis in whole liver tissue lysates. If indicated, rmIL‐1β protein (1 μg i.p.) was administered 30 min before the insulin injection. Data in A and B represent mean of three, respectively, two independent experiments performed in duplicate ± SEM. In C, representative immunoblots with densitometric analysis of three independent experiments are shown. *p < .05, **p < .01, ***p < .001 for insulin alone vs. insulin + rmIL‐1α/β and ^$^ *p* < .05, ^$$^ *p* < .01, ^$$$^ *p* < .001 for untreated versus treated using unpaired, two‐tailed Student's *t*‐test (A‐C)

Given that IL‐1 molecules can exert their effects directly on the hepatocytes contributing to alterations in hepatic metabolism and inflammation in the NAFL (Figure S4, S7, and S8), we examined whether IL‐1α/β – regardless of its cellular source – can also directly interfere with the insulin signalling pathway in hepatocytes as previously suggested.^39^ Primary WT hepatocytes were stimulated ex vivo with rmIL‐1α or rmIL‐1β protein and insulin (Figure 7A). While a tendency towards increased IRS‐1 mRNA expression was noted in response to insulin, simultaneous administration of rmIL‐1α or rmIL‐1β as well as prestimulation with rmIL‐1α/β significantly repressed gene expression of IRS‐1. Both insulin and rmIL‐1α/β led to a significant decrease in IRS‐2 mRNA after 3 h. Cotreatment slightly augmented these effects, while prestimulation with rmIL‐1α or rmIL‐1β over 18 h prevented downregulation of IRS‐2 from insulin, suggesting that chronic IL‐1 exposure can impair insulin signalling in hepatocytes.

Interestingly, inhibition of JNK1/2 using the small molecule inhibitor SP600125 did not prevent the downregulation of IRS‐1 and IRS‐2 expression induced by rmIL‐1β in insulin‐treated hepatocytes at 3 h (Figure 7B). In contrast, UO126 that abolished ERK1/2 activation prevented the decrease of IRS‐2 in rmIL‐1β‐stimulated hepatocytes and overcomes the inhibitory effect of rmIL‐1β on IRS‐2 in insulin‐treated hepatocytes (Figure 7B). Expression of IRS‐1 was unchanged by UO126. This suggests that the activation of the IL‐1R1 signalling pathway in metabolic liver disease interferes with insulin‐regulated gene transcription, whereby the effects on IRS‐2 – comparable to effects on PGC‐1α (Figure S7 and S8) – seem to be ERK1/2 dependent.

To evaluate the effects of IL‐1 signals on hepatic insulin signalling in vivo, naïve WT mice received an i.p. injection with either rmIL‐1β or vehicle (PBS) followed by an i.p. injection with insulin after 30 min (Figure 7C). Western blot analysis of liver tissue, harvested 10 min after insulin administration, revealed that pretreatment with rmIL‐1β blunted the extent of insulin‐induced IRS‐1 phosphorylation at Ser302 and AKT phosphorylation at Ser473 in mice. Thus, IL‐1β is capable of impairing insulin signalling and action in hepatocytes and thus could participate in the development of hepatic insulin resistance. Nevertheless, in insulin‐treated mice, blood glucose levels dropped to a greater extent following rmIL‐1β administration (data not shown), confirming an insulin‐independent, prolonged hypoglycemic effect of IL‐1β.^40^

### 3.8 Expression of IL‐1R1 in human disease

In human tissue, overall IL‐1R1 expression was faint in hepatocytes and more visible in immune cells – in particular macrophages – in the liver – with no apparent difference between NAFLD and NASH cases using immunohistochemistry (Figure S11A,B). Thus, although IL‐1‐mediated effects were reported in liver tissue under disease conditions, the surface protein expression level of IL‐1R1 in the liver was low with no significant increase in NASH. In vitro human primary hepatocytes upregulated transcripts in response to IL‐1 signalling (Figure S12A). We, therefore, next analysed human HepG2 cells for basal and IL‐1‐induced IL‐1R1 mRNA and protein expression. HepG2 cells displayed a continuous increase in IL‐1R1 transcript levels following IL‐1 stimulation in parallel to an upregulation of IL‐1RAcP (Figure S12B). At a functional level, IL‐1 signalling was inducing NF‐κB p65 activation detected by Western blot and ELISA assay (Figure S12C,D). Comparable to the findings in human NASH, IL‐1R1 protein expression remained unaffected by IL‐1 signals as shown by Western blot analysis (Figure S12E). These data indicate that IL‐1 signals – despite signal transduction – do not reconfigure the IL‐1R1 system by markedly increasing IL‐1R1 protein levels on hepatocytes and suggest that not receptor expression rather ligands are dysregulated in NAFLD.

## 4 DISCUSSION

Hepatic insulin resistance is a key feature in obesity and type 2 diabetes. In patients with NAFLD, insulin resistance and metabolic inflammation are associated with increased levels of circulating cytokines including IL‐1. The extent to which hepatocytes are involved in the regulation cytokine signalling, and if so, how this could be exploited as a therapeutic target remains undetermined.^37^ Our previous studies^16^ and publicly available single cell RNA‐Seq datasets as the comprehensive dataset of the Tabula Muris Consortium downloaded from the Single Cell Expression Atlas (https://www.ebi.ac.uk/gxa/sc/home)^18^ indicate that IL‐1R1 is indeed expressed in liver cells of naive mice, namely in liver sinusoidal endothelial cells, Kupffer cells, and hepatocytes (Table S2). This expression is even higher than in inflammatory cells such as granulocytes, monocytes, macrophages, or B cells. Single cell RNA‐Seq datasets of human samples, which show a high IL‐1R1 expression in liver sinusoidal endothelial cells and hepatic stellate cells, demonstrate moderate expression in hepatocytes and Kupffer cells and lower expression in liver resident inflammatory cells including plasma cells, B cells and NK cells (Table S3 and S4).^19^, ^20^ Therefore, we further investigated the specific role of the IL‐1R1 pathway in an HFD mouse model. As global knockout strategies have failed to detail tissue‐specific aspects of IL‐1 signalling, we developed a hepatocyte‐specific IL‐1R1 knockout model^17^ to study the initiating metabolic derangement in the hepatic compartment. Transgenic mice (naive, on CD) did not show a spontaneous metabolic phenotype as confirmed by global gene expression analysis (Figures S1 and S2 and Table S7). Using a HFD model of obesity and early‐stage NAFLD, the current study failed to detect an effect of hepatocellular IL‐1R1 on bodyweight or dyslipidaemia. However, marked improvement of insulin resistance, including improved hyperinsulinemia, HOMA‐IR, Adipo‐IR, and whole‐body glucose sensitivity were detectable in mice lacking IL‐1R1 in hepatocytes. Improvement of hepatic insulin sensitivity involved the regulation of insulin receptor, the intracellular signalling adapter IRS‐1/2 and downstream phosphorylation of the insulin‐sensitive kinase AKT at Ser473.

The adipose is a second important compartment that controls lipid and glucose homeostasis. In the visceral adipose tissue, we observed decreasing macrophage‐dominated inflammation and retained expression of IRS‐1/2 in mice with hepatocyte‐specific IL‐1R1. In this context, there is ample evidence that adipose‐initiated signals are capable to convey extra‐adipose tissue effects^8^, ^41^ and patients with obesity and type 2 diabetes have a pronounced proinflammatory signature in adipose tissue‐resistant macrophages driven by NLRP3‐dependent IL‐1β production.^42^ The current results underline a prominent role of IL‐1α and the cytokine antagonist IL‐1Ra but not IL‐1β in the adipose tissue during the early stage of NAFLD. This cytokine signature has also been observed in patients with obesity, insulin resistance, and the risk to develop type 2 diabetes.^43^ In the current study, we observed increased levels of circulating chemokines that are known to contribute to the recruitment of immune cells into adipose tissue and liver and perpetubate liver injury in NAFLD. The enhanced expression of CD206 indicated that a M2 polarization of intrahepatic macrophages occurred in the absence of hepatic IL‐1R1. Also, we observed substantial activation of JNK and ERK in the liver tissue of WT mice that was absent in *Il1r1* ^Hep−/–^ mice. JNK signalling has been shown to regulate hepatic insulin sensitivity in NAFLD^44^, ^45^ and compound deletion of JNK1 and JNK2 in the liver improved glucose homeostasis and insulin resistance involving increased hepatic fatty acid β‐oxidation.^46^ Moreover, JNK‐mediated autophagy has been linked to insulin resistance in the context of a HFD model in rats.^47^

Limitations of the current model must be acknowledged, as it produced only a mild inflammatory phenotype but prominent changes in insulin sensitivity. To partly overcome this limitation, we employed in vitro approaches to study the regulation of hepatic insulin signalling through IL‐1α and IL‐1β at the cellular level. Although metabolic profiling of cells in 2D culture systems often fails to reflect the metabolism occurring within tissues in vivo due to the lack of other cell types and 3D interaction, we could show that IL‐1α/β  decreased IRS‐1 independently of insulin and promoted insulin‐induced IRS‐2 degradation in primary hepatocyte cultures, thus interfering with insulin signalling. This was supported by our in vivo finding demonstrating that IL‐1β administration reduced insulin‐induced AKT phosphorylation in the liver, as previously described.^39^ Interestingly, the inhibitory effect of IL‐1α/β on IRS‐2, but not IRS‐1 was dependent on the duration of IL‐1 exposure and ERK1/2 signalling. In addition to MAPKs, IL‐1β was shown to enhance the SOCS‐mediated degradation of IRS‐1/2 involving the IkappaB kinase beta (IKKβ)/NF‐κB pathway, promoting glucose intolerance and hepatic insulin resistance by this mechanism.^48^, ^49^ However, although hepatocyte‐specific knockout of *Il1r1* showed increased basal levels of hepatic SOCS3, we did not observe a spontaneous phenotype of insulin resistance or other metabolic perturbances in the knockout mice.

Albeit retaining an insulin‐sensitive state, HFD‐fed *Il1r1* ^Hep−/–^ mice developed macrovesicular hepatic steatosis. Marked differences between the WT and IL‐1R1 knockout were only seen at the level of microvesicular steatosis that was significantly less pronounced in the *Il1r1* ^Hep−/–^ mice on HFD. Microvesicular steatosis is linked to impaired mitochondrial β‐oxidation of fatty acids and increased oxidative damage. Reduced mitochondrial function compromises overall cellular oxidative capacity leading to the accumulation of lipotoxic lipid intermediates and favouring of insulin resistance, which is exacerbated by impaired autophagy.^50^ Gene and protein expression analysis revealed that *Il1r1* ^Hep−/–^ mice fed with the HFD exhibited an increased hepatic expression of PGC‐1α, a key transcriptional regulator of mitochondrial metabolism and energy homeostasis. Importantly, reductions of hepatic PGC‐1α are associated with insulin resistance in type 2 diabetes and NAFLD in humans.^51^, ^52^ Mouse models showed that physiological reductions of PGC‐1α disrupt insulin signalling in liver,^53^ and mice with low hepatic PGC‐1α are more susceptible to hepatic steatosis, hypertriglyceridaemia, and oxidative liver damage – hallmarks of insulin resistance.^17^, ^53^ In contrast, overexpression of hepatic PGC‐1α and subsequent increases in fatty acid β‐oxidation through elevated mitochondrial content and/or function resulted in reduced triglyceride storage and secretion in vitro and in vivo and disposing of potentially injurious lipid species.^54^ Only a few studies have linked hepatic PGC‐1α to the inappropriate activation of gluconeogenesis in type 2 diabetes.^55^ The antidiabetic drug metformin acts directly on mitochondria and affects the expression and function of hepatic PGC‐1α.^56^ In this context, Lustig et al. demonstrated that S6K1, a serine/threonine kinase that mediates nutrient and insulin signals, is able to separate the gluconeogenic and mitochondrial functions of PGC‐1α through direct phosphorylation.^57^ Importantly, differentially spliced variants of the PGC‐1α protein have unique functions in regulating hepatocyte responses to concurrently integrate metabolic and inflammatory signals facilitating parallel adaptions to metabolic demands and mitigation of inflammatory damage in hepatocytes.^58^ Along this line of evidence, we observed that HFD‐fed *Il1r1* ^Hep−/–^ mice exhibited a restoration of NRF‐1 and TFAM expression levels, which are also closely associated with the regulation of mitochondrial function. Disruption of the NRF‐1‐TFAM pathway was recently shown to contribute to mitochondrial dysfunction and liver damage in alcoholic steatohepatitis.^59^

Additionally, HFD‐fed *Il1r1* ^Hep−/–^ mice exhibited a higher hepatic expression of the PGC‐1α interaction partner FXR‐α as well as FXR−α target genes including CES‐1 compared to WT littermates. Through this, adverse effects of HFD on glucose and lipid homeostasis, mitochondrial dysfunction and hepatic insulin signalling can be ameloriated.^33^ In addition, FXR‐α activity was shown to suppress NF‐κB agonist‐induced inflammatory gene expression in an FXR‐α‐dependent manner in HepG2 cells and mouse primary hepatocytes^60^ as well as CRP release,^61^ suggesting that blocking IL‐1R1 in hepatocytes prevents dysregulation of both PGC‐1α and FXR‐α in NAFLD. Our in vitro studies confirmed that IL‐1α/β treatment significantly reduces PGC‐1α and FXR‐α mRNA levels in the human hepatoma cell line HepG2, in human and mouse primary hepatocytes, as previously reported.^62^ This regulation was ERK1/2 dependent. Interestingly, FXR ligands have also been shown to protect against hepatocellular inflammation through induction of SOCS3,^63^ and SOCS3 levels were retained in *Il1r1* ^Hep−/–^ but not the WT mice after HFD feeding. Disruption of IL‐1R1 signalling was associated with moderate SIRT1 induction and an increase in LC3B II protein levels under HFD conditions,^64^ increasing the liver capacity of *Il1r1* ^Hep−/–^ mice to oxidize lipids and to activate autophagy processes, thus preventing steatosis aggravation and insulin resistance. Decreased autophagy was also thought to be a mechanism for IL‐1β‐dependent hepatic injury in NAFLD,^65^ which might explain the correlation between the reduced LC3B II levels and increased hepatic injury in WT compared to *Il1r1* ^Hep−/–^ livers under steatotic conditions.

Despite this critical role for IL‐1/IL‐1R1 signalling in hepatocytes during the early stage of NAFLD, we could not observe a dysregulation of IL‐1R1 protein expression on hepatocytes in human NAFLD. The faint IL‐1R1 staining in liver tissue was in line with a study using an IL‐1R1 reporter mouse model, which could not detect a positive signal in hepatic tissue.^66^ Importantly, this does not exclude the functionality of the IL‐1R1 pathway and that blocking the IL‐1R1 in hepatocytes has beneficial effects on certain aspects of metabolic liver disease.

In conclusion, our study provides evidence that hepatocellular IL‐1 signalling is directly involved in glucose homeostasis and adipose tissue inflammation through MAPKs and downstream effectors. Thus, hepatocyte‐directed IL‐1 inhibition could be an adjunctive strategy to weight loss in obese patients to improve hepatic insulin resistance, mitochondrial dysfunction, and ultimately the risk to develop overt type 2 diabetes.

## CONFLICT OF INTEREST

JMS declares consultant honorary from BMS, Boehringer Ingelheim, Echosens, Genfit, Gilead Sciences, Intercept Pharmaceuticals, Madrigal, Merck, Nordic Bioscience, Novartis, Pfizer, Roche, Sanofi, and Siemens Healthcare GmbH, research funding from Gilead Sciences, Boehringer Ingelheim, Siemens Healthcare GmbH, and speaker honorarium form Falk Foundation. The other authors declare no conflict of interest.

## Supporting information

Supporting Information

## Data Availability

Data available on request from the authors.
