# Supplementary material for: Hepatic interleukin‐1 receptor type 1 signalling regulates insulin sensitivity in the early phases of nonalcoholic fatty liver disease
Source: Clin Transl Med. 2022 Sep 13;12(9):e1048. doi: 10.1002/ctm2.1048 (PMC9471277; doi:10.1002/ctm2.1048)
Supplement: Supplementary file 1 — Supporting Information [file CTM2-12-e1048-s001.docx]

**Supplementary Data**

**Hepatic interleukin-1 receptor type 1 signalling regulates insulin sensitivity in the early phases of nonalcoholic fatty liver disease**

Nadine Gehrke, Lea J. Hofmann, Beate K. Straub, Frank Rühle, Ari Waisman, Peter R. Galle, Jörn M. Schattenberg

**Table of content**

**Supplementary Figures - Results3**

Supplementary Figure 1: Body weight, glucose tolerance and insulin sensitivity in naïve *Il1r1*^Hep-/-^ and WT mice. 3

Supplementary Figure 2: Liver transcriptome analysis and immunoblotting reveal differential SOCS3 expression between *Il1r1*^Hep-/-^ and WT mice.4

Supplementary Figure 3: Rate of fatty acid-induced hepatocyte cell death *in vitro*. 5

Supplementary Figure 4: IL-1-induced effects on lipid metabolism associated genes in human HepG2 cells and primary hepatocytes. 6

Supplementary Figure 5: Regulation of lipid metabolism-related gene expression in the liver of *Il1r1*^Hep-/-^ and WT mice in response to HFD feeding. 7

Supplementary Figure 6: Differential hepatic expression of SIRT1 and the autophagy-related protein LC3B in *Il1r1*^Hep-/-^ vs. WT mice fed the HFD. 8

Supplementary Figure 7: IL-1 signals repress PGC-1α and FXR-α expression in primary murine hepatocytes and are potent inducers of the neutrophil-attractant chemokines CXCL-1 and CXCL-2. 9

Supplementary Figure 8: Treatment of human HepG2 cells and primary human hepatocytes with IL-1α/β rapidly induces suppression of PGC-1α and FXR-α expression and IL-8 upregulation. 10

Supplementary Figure 9: Insulin signalling and glucose uptake in the adipose tissue of HFD-fed *Il1r1*^Hep-/-^ and WT mice. 11

Supplementary Figure 10: Metabolic HFD-challenge resulted in an early stage of NAFLD and concomitant insulin resistance in the absence of significant hepatic inflammation. 12

Supplementary Figure 11: Immunohistochemical analysis of IL-1R1 expression in human NAFLD. 14

Supplementary Figure 12: IL-1R1 protein expression remains unchanged in human hepatocytes in response to IL-1 signalling despite enhanced transcription. 15

**Supplementary Tables – Material and Methods17**

Supplementary Table 1: Crude nutrients of experimental diets (both ssniff Spezialdiäten GmbH, Soest, Germany). .17

Supplementary Table 2: IL-1R1 expression in cells of the Tabula Muris compendium. 18

Supplementary Table 3: IL-1R1 expression in human liver cells (data set from Wang et al).25

Supplementary Table 4: IL-1R1 expression in human liver cells (data set from MacParland et al). 26

Supplementary Table 5: Mouse forward and reverse primers used for qRT-PCR. 27

Supplementary Table 6: Human forward and reverse primers used for qRT-PCR. 29

**Supplementary Tables – Results30**

Supplementary Table 7: Significantly differentially expressed genes from comparison of naïve, male *Il1r1*^Hep-/-^ vs. WT mice at 10 weeks of age (n=6 mice/genotype). 30

**Supplementary Figures – Results**

**Supplementary Figure 1: Body weight, glucose tolerance and insulin sensitivity in naïve *Il1r1*^Hep-/-^ and WT mice.** (A) Body weight curves, (B) i.p. glucose tolerance test, and (C) insulin-stimulated hepatic AKT activation (at 15 minutes) in naïve, 6-mo-old, male *Il1r1*^Hep-/-^ mice and WT littermates. Data in A represent mean of n=17 *Il1r1*^Hep-/-^ and n=9 WT, in B n=13 *Il1r1*^Hep-/-^ and n=11 WT mice ± SEM. In C a representative immunoblot with densitometric analysis of three independent experiments is shown. There was no statistically significant difference between the two groups with respect of the parameters (A-C).


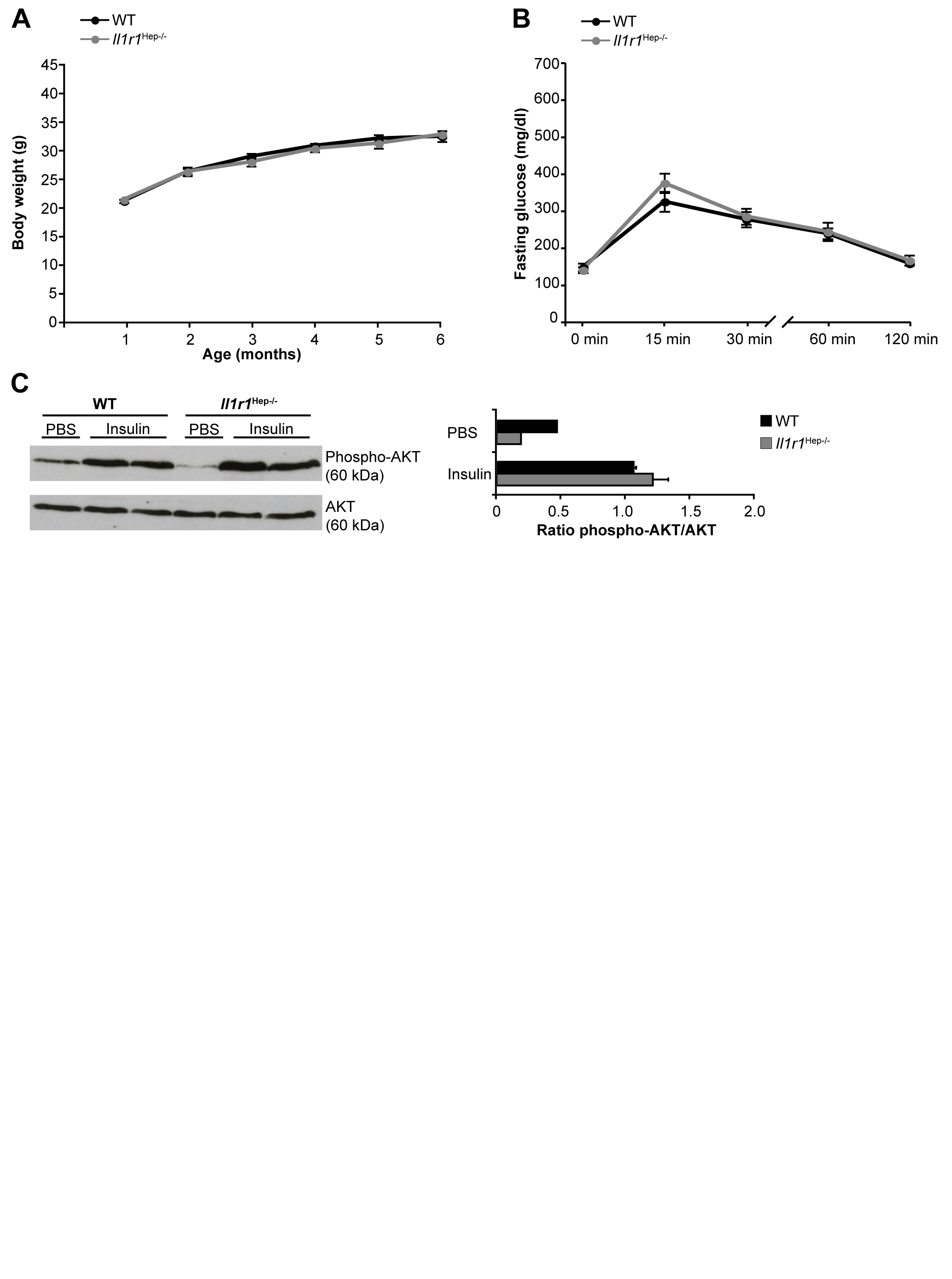


**Supplementary Figure 2: Liver transcriptome analysis and immunoblotting reveal differential SOCS3 expression between *Il1r1*^Hep-/-^ and WT mice.** (A) Volcano plot of all DESeq2-tested genes depicting their expression fold change vs. statistical significance (naive *Il1r1*^Hep-/-^ vs. WT mice at 10 weeks of age, n=6 mice/genotype). The FDR < 0.05 hits are highlighted in red and labelled. Mean normalized counts (log10 transformed) are indicated by symbol size. (B) Immunoblotting of SOCS3 in liver whole tissue lysates from *Il1r1*^Hep-/-^ and WT mice fed the HFD or the CD for 12 weeks. α-Tubulin served as protein loading control. A representative immunoblot with densitometric analysis is shown. * p<0.05 for *Il1r1*^Hep-/-^ vs. WT using unpaired, two-tailed Student’s *t*-test (B).


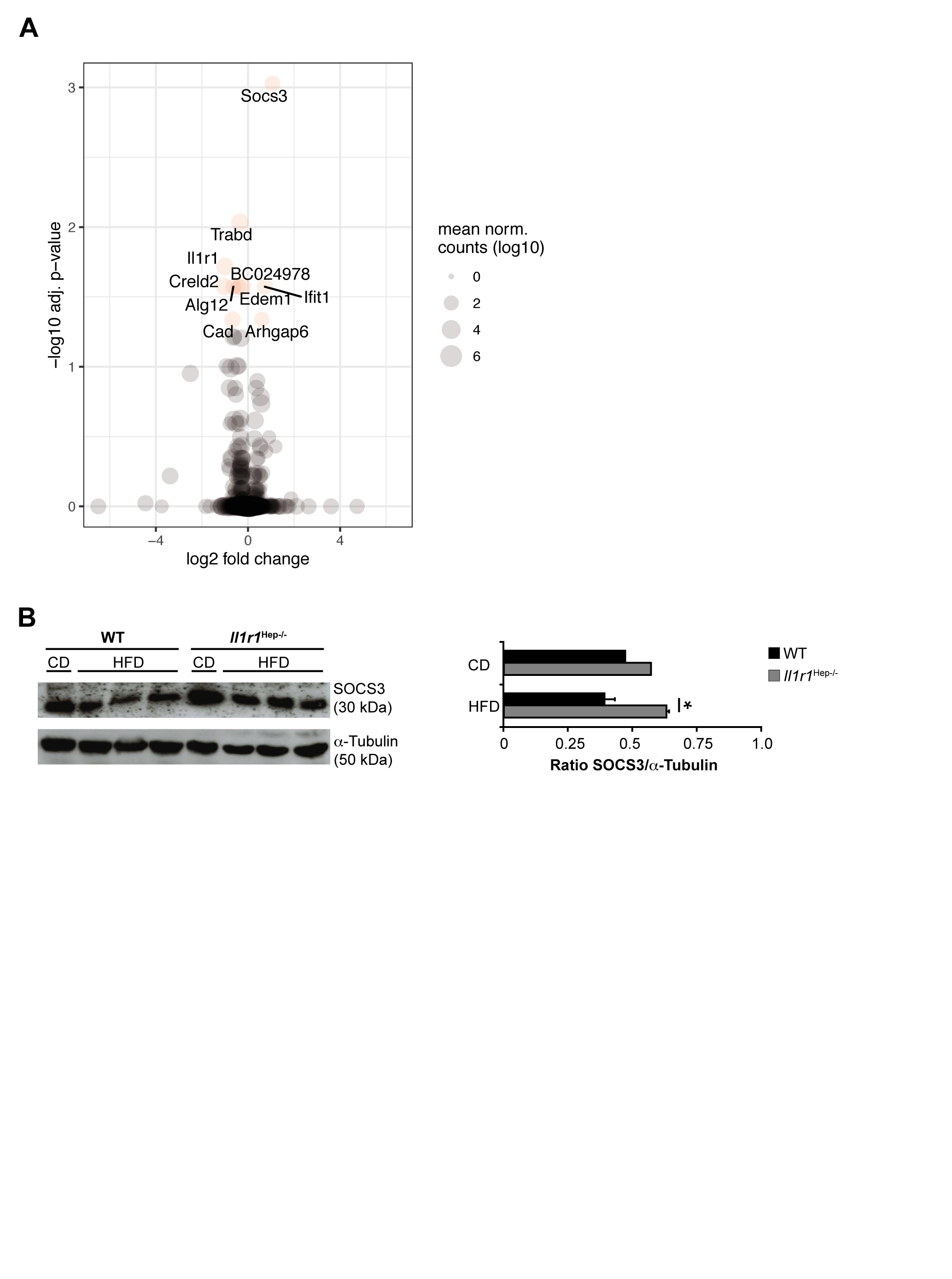


**Supplementary Figure 3: Rate of fatty acid-induced hepatocyte cell death *in vitro.*** Primary WT hepatocytes were treated *ex vivo* with different concentrations of (A) palmitic acid resp. (B) oleic acid in the absence or presence of rmIL-1α/β protein (10 ng/ml). After 24 h cell viability was assessed by MTT colorimetric assay relative to untreated samples. Numerical data in mean ± SEM of three independent experiments performed in at least duplicate readings. * p<0.05, ** p<0.01 for fatty acid alone vs. fatty acid + rmIL-1α/β and ^$$^ p<0.01, ^$$$^ p<0.001 for untreated vs. treated hepatocytes according to an unpaired, two-tailed Student’s t-test (A and B).


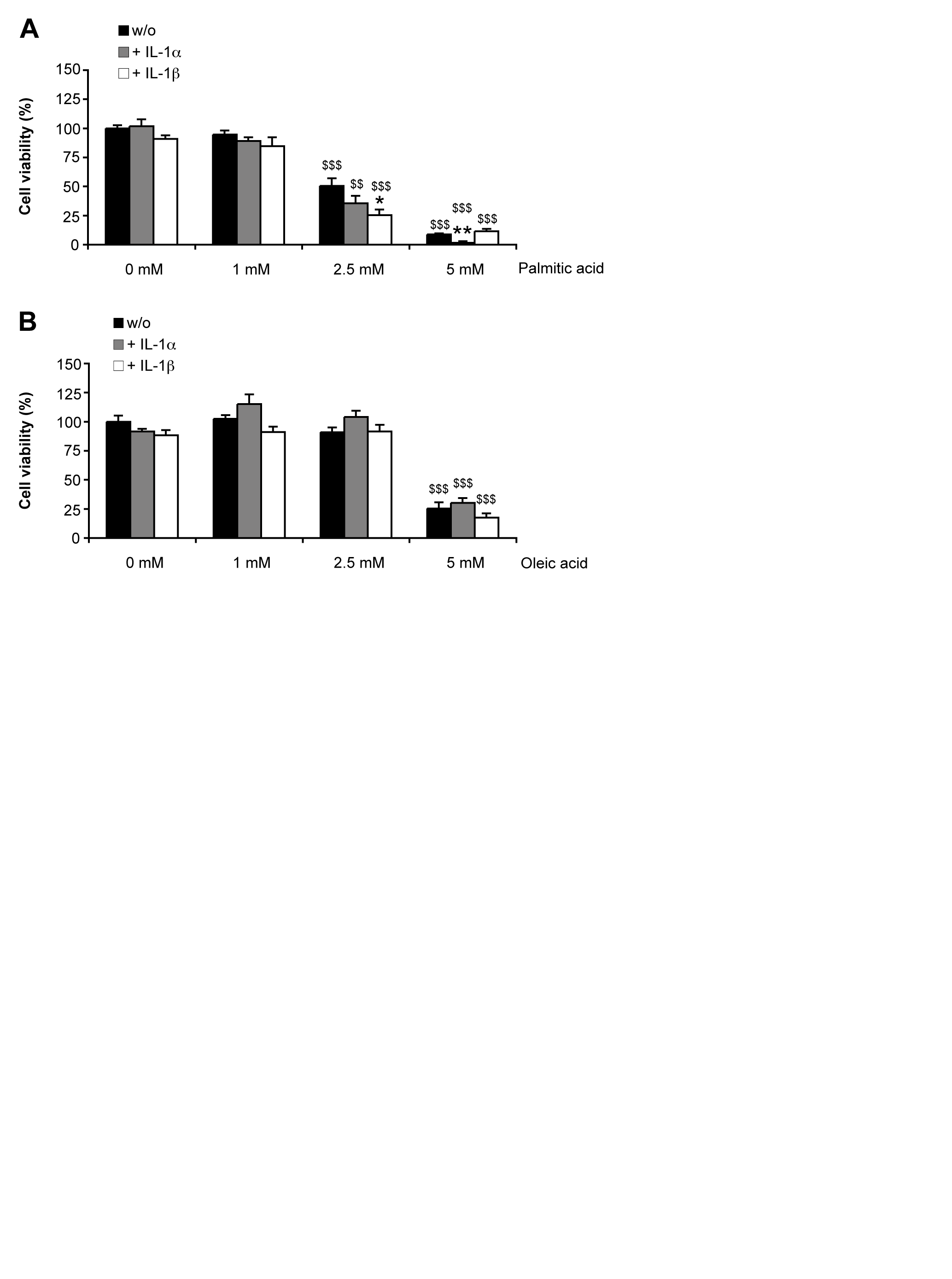


**Supplementary Figure 4: IL-1-induced effects on lipid metabolism involved genes in human HepG2 cells and primary hepatocytes.** Relative mRNA expression analysis of SREBP-1c, PPAR-α and CPT1 (encoded by *SREBF1*, *PPARA*, resp. *CPT1A*) using qRT-PCR in human HepG2 cells (A) and primary human hepatocytes (B and C) following 3-h-treatment with rhIL-1α/β (100 ng/ml). In A and C transcript levels were also determined in BSA-Oleate-induced (200 µM, 24 h) steatotic cells. * p<0.05, ** p<0.01, ** p<0.001 for untreated vs. treated cells according to an unpaired, two-tailed Student’s t-test (A-C).

**
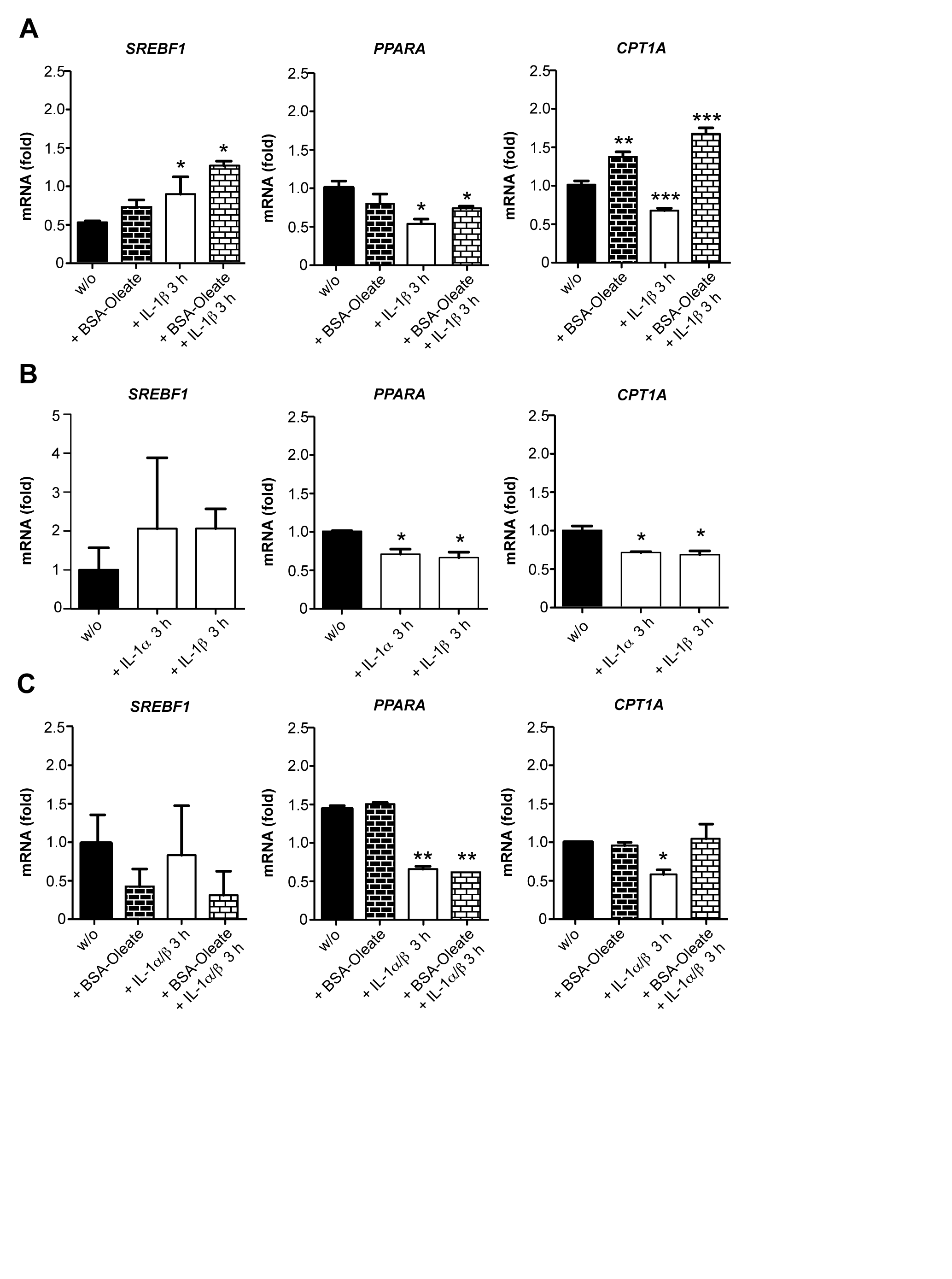
**

**Supplementary Figure 5: Regulation of lipid-metabolism involved genes in the liver of *Il1r1*^Hep-/-^ and WT mice in response to HFD feeding.** Relative mRNA expression of key regulators of hepatic lipid metabolism determined by qRT-PCR analyses in liver whole tissue lysates from *Il1r1*^Hep-/-^ and WT mice fed the HFD or the CD for 12 weeks. Data represent mean of n=4 *Il1r1*^Hep-/-^ CD, n=4 WT CD, n=7 *Il1r1*^Hep-/-^ HFD and n=7 WT HFD mice ± SEM. ^$^ p<0.05, ^$$^ p<0.01 for CD vs. HFD using two-way method of ANOVA following the Bonferroni multiple comparison tests.


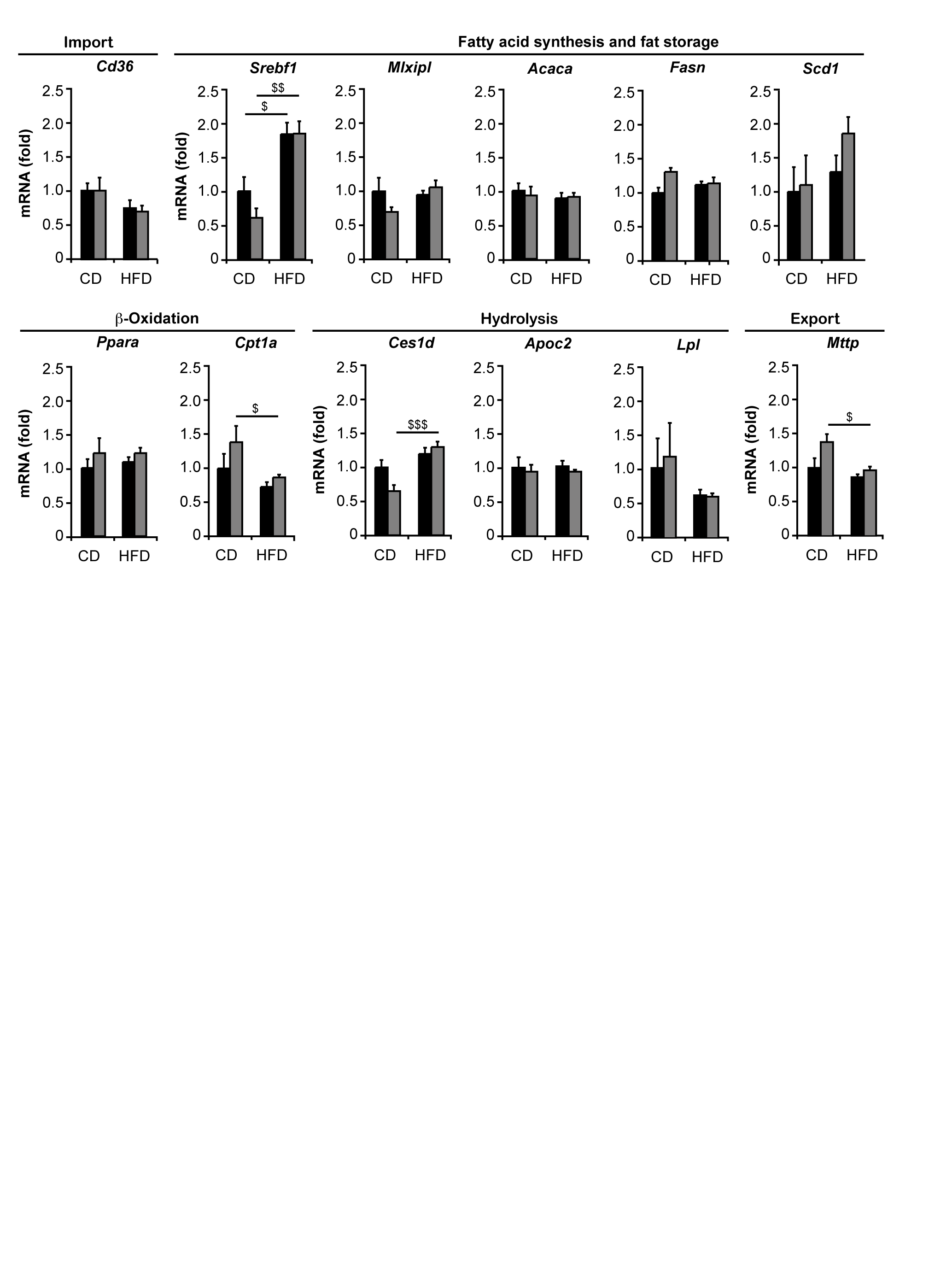


**Supplementary Figure 6: Differential hepatic expression of SIRT1 and the autophagy-related protein LC3B in *Il1r1*^Hep-/-^ vs. WT mice fed the HFD.** Total liver tissue lysates from the different experimental groups were analyzed for (A) SIRT1 mRNA and (B) LC3B-II protein levels. Protein bands were quantified and normalized to α-Tubulin expression as fold change over CD. Data in A represent mean of n=4 *Il1r1*^Hep-/-^ CD, n=4 WT CD, n=7 *Il1r1*^Hep-/-^ HFD and n=7 WT HFD mice ± SEM. There was no statistically significant difference between the experimental groups. In B a representative immunoblot with densitometric analysis is shown. * p<0.05 for *Il1r1*^Hep-/-^ vs. WT using unpaired, two-tailed Student’s *t*-test (B).

**
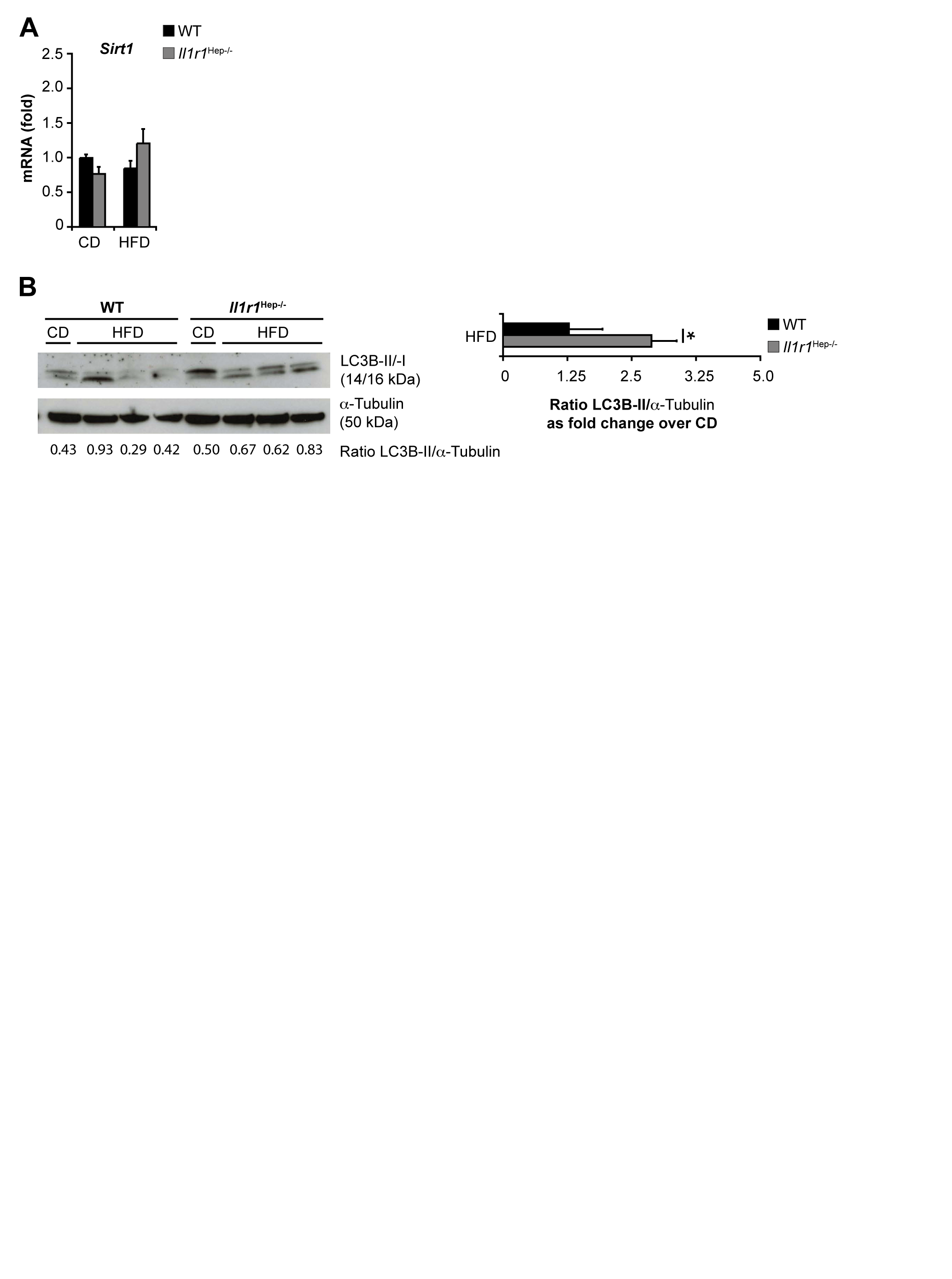
**

**Supplementary Figure 7: IL-1 signals repress PGC-1α and FXR-α expression in primary murine hepatocytes and are potent inducers of the neutrophil-attractant chemokines CXCL-1 and CXCL-2.** Relative mRNA expression of (A) PGC-1α, (B) FXR-α, (C) CXCL-1, and (D) CXCL-2 in primary WT hepatocytes following e*x vivo* 3-h- and 18-h-treatment with rmIL-1α or rmIL-1β protein (10 ng/ml). Data in A-D represent mean of three independent experiments performed in duplicate ± SEM. * p<0.05, ** p<0.01, *** p<0.001 for untreated vs. IL-1α/β-treated cells using unpaired, two-tailed Student’s *t*-test (A-D).


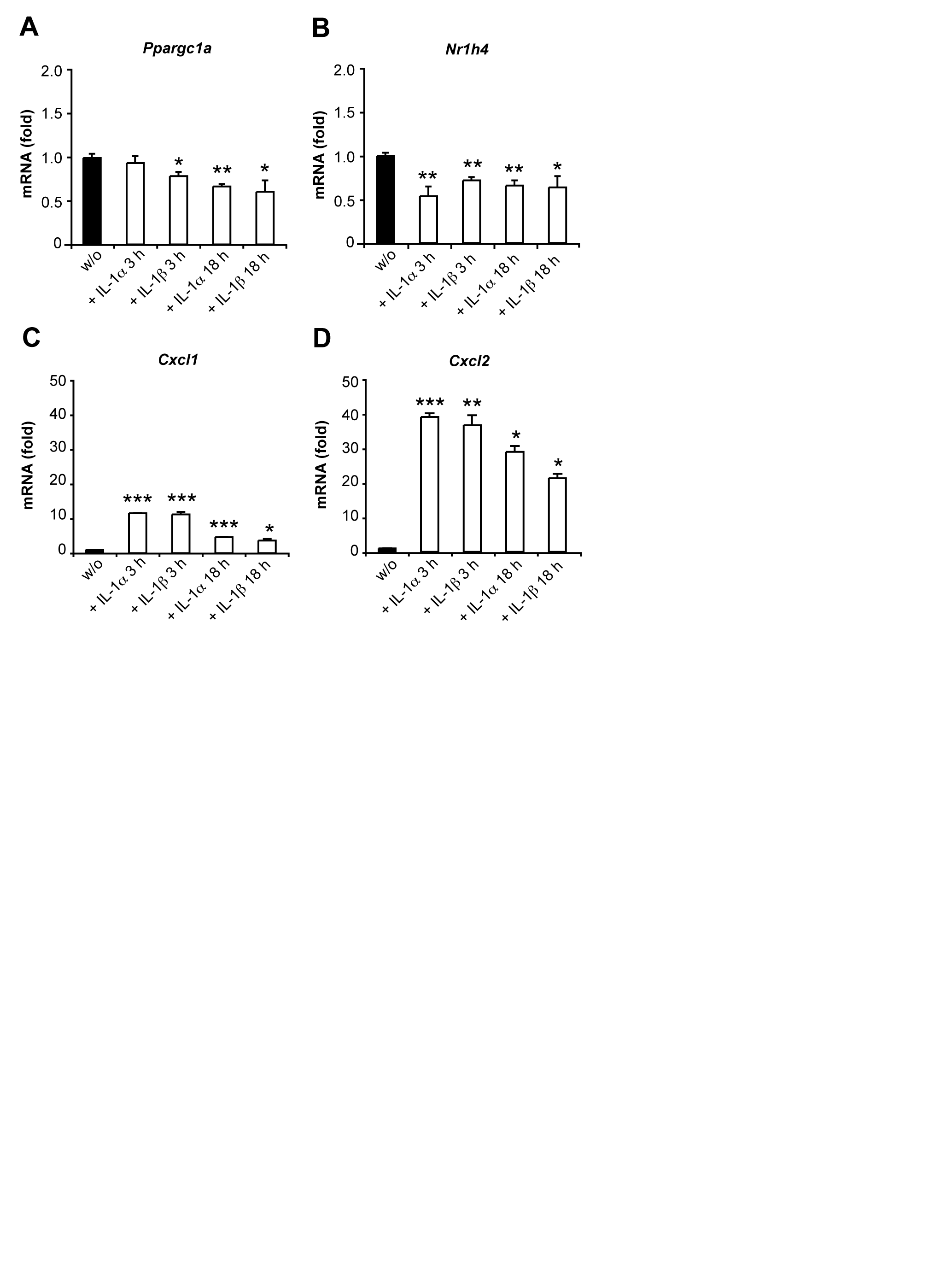


**Supplementary Figure 8: Treatment of human HepG2 cells and primary human hepatocytes with IL-1β rapidly induces suppression of PGC-1α and FXR-α expression and IL-8 upregulation.** Gene expression analysis in HepG2 cells (A, C and D) and human primary hepatocytes (B and E) was performed at 3 h post stimulation with rhIL-1β protein (100 ng/ml). In A and D the NF-κB inhibitor BAY-11-7082 (10 µM), the JNK inhibitor SP600125 (100 µM), or the ERK inhibitor UO126 (50 µM) was added to cultures 1 h prior to stimulation. In C transcript levels were determined also in BSA-Oleate-induced (200 µM, 24 h) steatotic HepG2 cells. Data in A-E represent one representative duplicate experiment out of two ± SEM. * p<0.05, * p<0.01 for untreated vs. IL-1β-treated cells and ^$^ p<0.05 for IL-1β-treated vs. IL-1β-treated post inhibitor pretreatment using unpaired, two-tailed Student’s *t*-test (A-E).


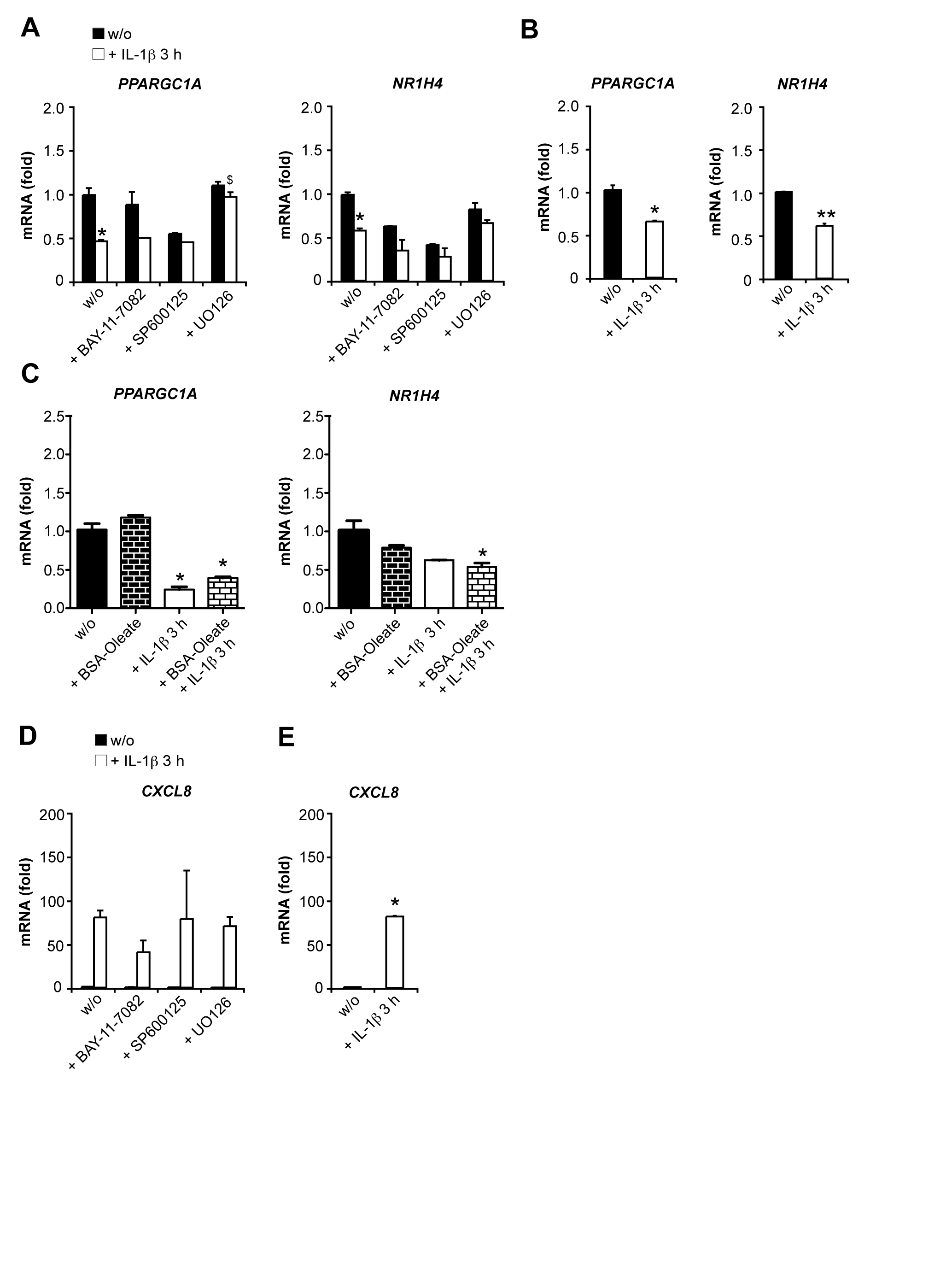
**Supplementary Figure 9: Insulin signalling and glucose uptake in the adipose tissue of HFD-fed *Il1r1*^Hep-/-^ and WT mice.** qRT-PCR assay of adipose tissue IRS-1, IRS-2 and GLUT4 mRNA expression in the different experimental groups after 12-week-feeding. Data represent mean of n=5 *Il1r1*^Hep-/-^ CD, n=4 WT CD, n=8 *Il1r1*^Hep-/-^ HFD and n=8 WT HFD mice ± SEM. ^$^ p<0.05 for CD vs. HFD using two-way method of ANOVA following the Bonferroni multiple comparison tests.


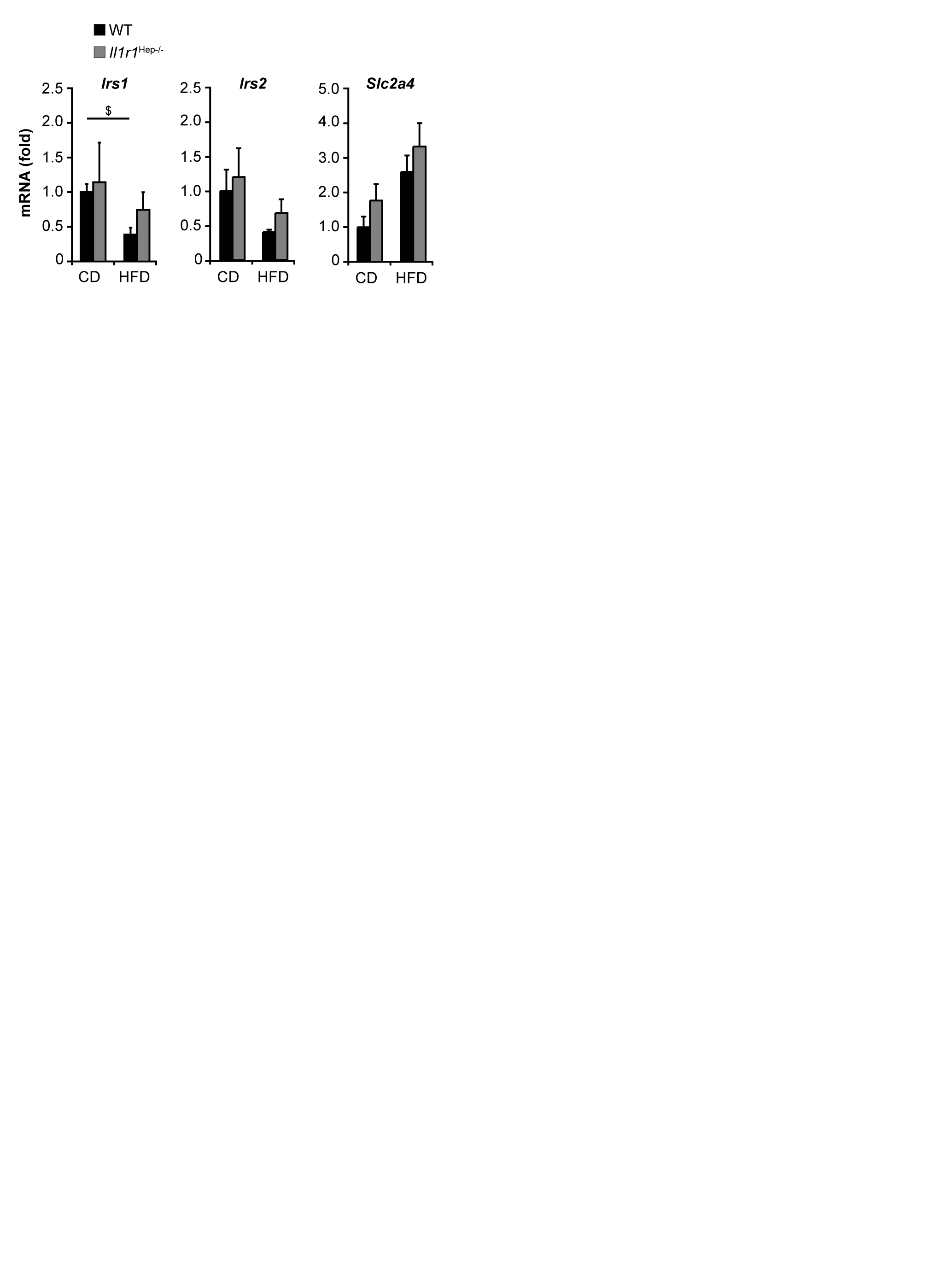


**Supplementary Figure 10: Metabolic HFD-challenge resulted in an early stage of NAFLD and concomitant insulin resistance in the absence of significant hepatic inflammation.** (A) Relative hepatic mRNA expression of cytokines and chemokines determined by qRT-PCR analysis, (B) relative proportions of various intrahepatic CD45^+^ immune cell subsets quantified by FACS, (C) measurement of CXCL-1 protein in serum using ELISA, and (D) qRT-PCR assay of hepatic CD206 and ARG1 mRNA expression in the different experimental groups after 12-week-feeding. Data in A-D represent mean of n=4 *Il1r1*^Hep-/-^ CD, n=4 WT CD, n=7 *Il1r1*^Hep-/-^ HFD and n=7 WT HFD mice ± SEM. * p<0.05 for *Il1r1*^Hep-/-^ vs. WT and ^$^ p<0.05, ^$$^ p<0.01 for CD vs. HFD using two-way method of ANOVA following the Bonferroni multiple comparison tests.(A-C). In D there was no statistically significant difference between the experimental groups, albeit *Mrc1* levels were generally higher in the *Il1r1*^Hep-/-^ transgenic livers (Diet: n.s., Gene: p<0.05, Interaction: n.s.).


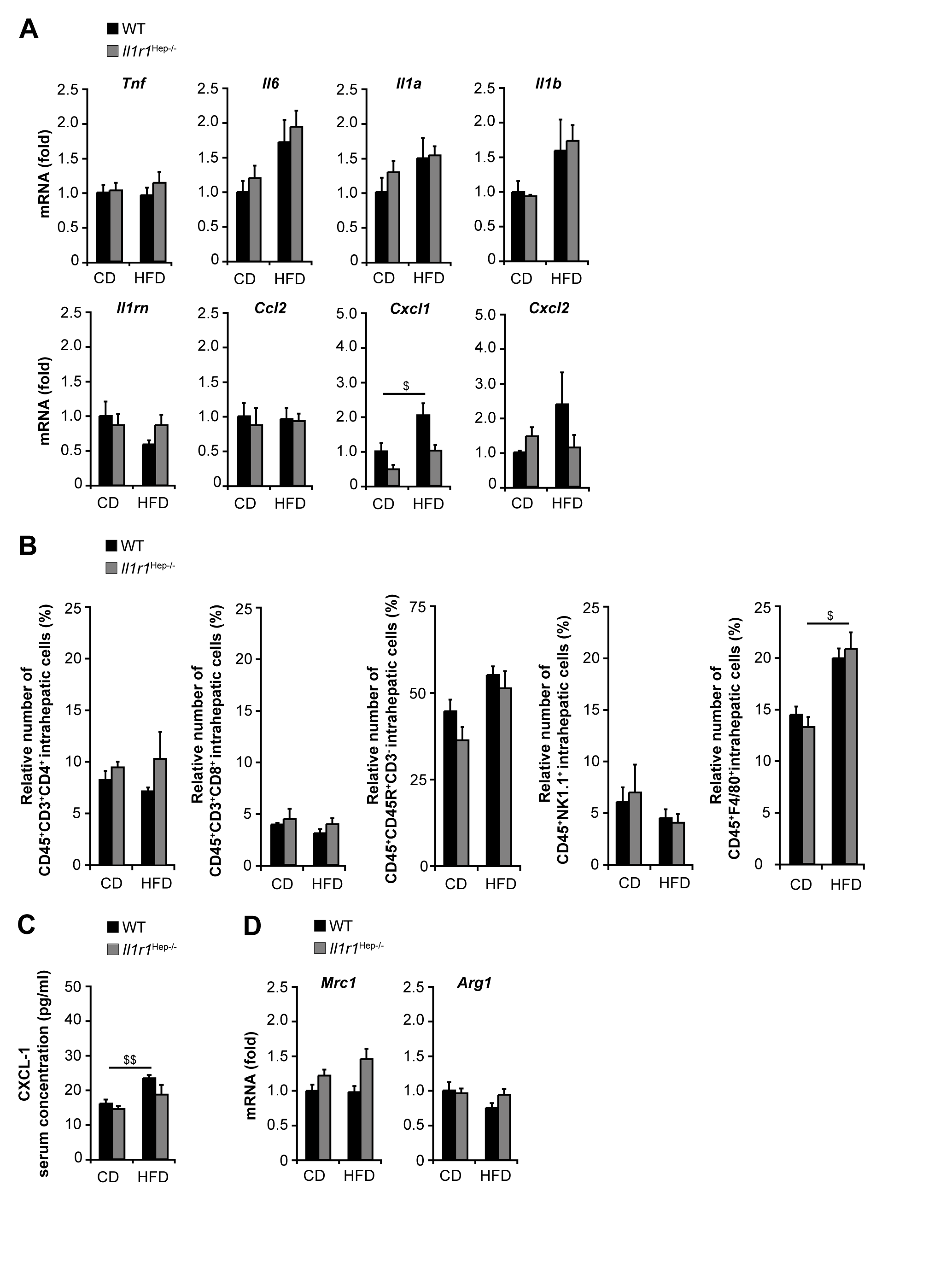


**Supplementary Figure 11: Immunohistochemical analysis of IL-1R1 expression in human NAFLD.** Immunohistochemical staining for IL-1R1 was performed in paraffin-embedded human liver tissue sections from patients with simple hepatic steatosis (NAS < 3) and NASH (NAS > 5), matched in age and sex, using an antibody from Novus Biologicals (Littleton, CO, USA) (A). Alternatively, an antibody from Sigma-Aldrich (St. Louis, MI, USA) was tested (B). Representative pictures are shown (scale bar: 50 µm). Although IL-1-mediated effects were reported in liver tissue under disease conditions, the general protein expression level of IL-1R1 in the human liver seems to be low - with immune cells as the highest IL-1R1-expressing cells.


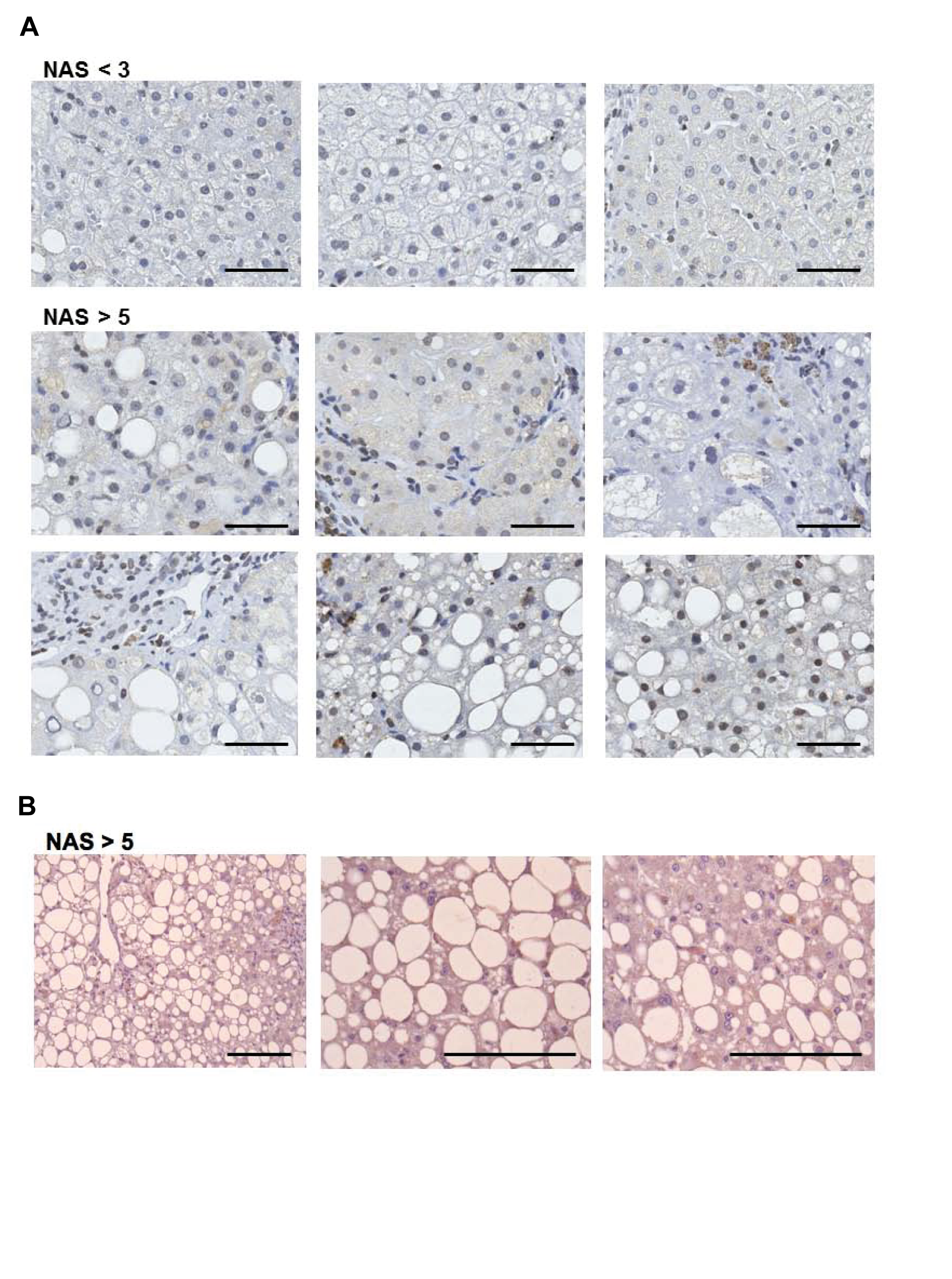


**Supplementary Figure 12: IL-1R1 protein expression remains unchanged in human hepatocytes in response to IL-1 signalling despite enhanced IL-1R1 gene transcription.**

(A) Relative mRNA expression of IL-1R1 in primary human hepatocytes, (B) IL-1R1 and IL-1RAcP in HepG2 cells following 3-h- and 18-h-treatment with rhIL-1α or rhIL-1β protein (100 ng/ml). (C) Western Blot and (D) ELISA analysis of phosphorylated (Ser536)/active NF-κB p65 protein in nuclear extracts of HepG2 cells at various times following IL-1β-stimulation. Lamin B1 served as protein loading control. (E) Representative western blot analysis of IL-1R1 expression in HepG2 cells following 18-h-treatment with rhIL-1α or rhIL-1β protein. α-Tubulin served as protein loading control. No increase in the expression of IL-1R1 protein was detected. Data in A and B represent one representative duplicate experiment out of two ± SEM. * p<0.05, * p<0.01 for untreated vs. IL-1-treated cells using unpaired, two-tailed Student’s *t*-test (A and B). In C and D a representative immunoblot and the corresponding ELISA assay data are shown.

**Supplementary Tables - Material and Methods**:

**Supplementary Table 1: Crude nutrients of experimental diets (both ssniff Spezialdiäten GmbH, Soest, Germany).**

| **Product No.** |  | **Surwit Diet (HFD)**  **[HF/sucrose]**  **E15772-34** | **Control Diet (CD)**  **[soybean oil]**  **E15772-04** |
| --- | --- | --- | --- |
| Casein | % | 23.00 | 23.00 |
| Corn stach, pre-gelatinized | % | - | 27.00 |
| Maltodextrin | % | 15.30 | 15.30 |
| Sucrose | % | 17.00 | 17.00 |
| Pur. Cellulose powder | % | 1.80 | 5.20 |
| DL-Methionine | % | 0.10 | 0.10 |
| L-Cystine | % | 0.10 | 0.10 |
| Mineral & trace element premix | % | 5.50 | 5.50 |
| Vitamin premix | % | 1.00 | 1.00 |
| Sodium phosphate, dibasic | % | 0.30 | 0.30 |
| Dye, red-yellow mix | % | 0.10 | - |
| Choline chloride | % | 0.20 | 0.20 |
| Coconut oil, hydrogenated | % | 33.30 | - |
| Soybean oil | % | 2.30 | 5.30 |
| ME, Atwater* | MJ/kg | 22.80 | 15.70 |
| Protein | kJ% | 15 | 22 |
| Carbohydrates | kJ% | 26 | 65 |
| Lipids | kJ% | 58 | 13 |

* physiological fuel value**Supplementary Table 2: IL-1R1 expression in cells of the Tabula Muris compendium.** Normalized expression counts were downloaded from the Single Cell Expression Atlas (<https://www.ebi.ac.uk/gxa/sc/experiments/E-ENAD-15/results/tsne>)^18^ and summarized (mean and standard deviation (SD)) by annotated organism part and cell type.

| **Organism part** | **Cell type** | **Mean**  ***Il1r1*** | **SD**  ***Il1r1*** |
| --- | --- | --- | --- |
| aorta | endothelial cell | 27.04 | 127.92 |
| aorta | erythrocyte | 2.61 | 16.55 |
| aorta | fibroblast | 110.1 | 221.42 |
| aorta | not assigned | 40.4 | 675.39 |
| aorta | professional antigen presenting cell | 9.33 | 53.65 |
| ascending colon | Brush cell of epithelium proper of large intestine | 6.81 | 47.52 |
| ascending colon | enterocyte of epithelium of large intestine | 4.86 | 39.12 |
| ascending colon | enteroendocrine cell | 17.33 | 54.64 |
| ascending colon | epithelial cell of large intestine | 21.83 | 55.96 |
| ascending colon | large intestine goblet cell | 34.22 | 80.75 |
| ascending colon | not assigned | 0 | 0 |
| back skin | basal cell of epidermis | 41.33 | 187.85 |
| back skin | epidermal cell | 35.01 | 65.45 |
| back skin | keratinocyte stem cell | 13.96 | 55.94 |
| back skin | leukocyte | 16.16 | 48.85 |
| back skin | not assigned | 34.05 | 188.75 |
| back skin | stem cell of epidermis | 27.88 | 49.7 |
| bone marrow | B cell | 1.02 | 3.95 |
| bone marrow | basophil | 0.68 | 1.92 |
| bone marrow | common lymphoid progenitor | 40.12 | 78 |
| bone marrow | granulocyte | 1.12 | 17.26 |
| bone marrow | granulocyte monocyte progenitor cell | 6.12 | 21.46 |
| bone marrow | hematopoietic precursor cell | 23.66 | 76.75 |
| bone marrow | immature B cell | 0.49 | 3.27 |
| bone marrow | immature natural killer cell | 0 | 0 |
| bone marrow | immature NK T cell | 1.2 | 6.45 |
| bone marrow | immature T cell | 0.72 | 5.55 |
| bone marrow | late pro-B cell | 0.58 | 2.8 |
| bone marrow | macrophage | 0.53 | 2.75 |
| bone marrow | mature natural killer cell | 0.58 | 2.84 |
| bone marrow | megakaryocyte-erythroid progenitor cell | 8.03 | 45.17 |
| bone marrow | monocyte | 0.42 | 4.24 |
| bone marrow | naive B cell | 0.52 | 8.5 |
| bone marrow | not assigned | 24.78 | 197.87 |
| bone marrow | pre-natural killer cell | 0.92 | 3.09 |
| bone marrow | pro-B cell | 1.37 | 8.28 |
| bone marrow | regulatory T cell | 10.2 | 53.01 |
| bone marrow | Slamf1-negative multipotent progenitor cell | 40.75 | 106.73 |
| bone marrow | Slamf1-positive multipotent progenitor cell | 25.34 | 50.13 |
| cerebellum | astrocyte of the cerebral cortex | 0.43 | 1.21 |
| cerebellum | Bergmann glial cell | 0.19 | 1.17 |
| cerebellum | brain pericyte | 66.66 | 247.76 |
| cerebellum | endothelial cell | 52.33 | 197.98 |
| cerebellum | macrophage | 0.75 | 3.62 |
| cerebellum | microglial cell | 2.08 | 25.52 |
| cerebellum | neuron | 24.66 | 198.02 |
| cerebellum | not assigned | 2.92 | 63.35 |
| cerebellum | oligodendrocyte | 0.96 | 9.63 |
| cerebellum | oligodendrocyte precursor cell | 3.82 | 13.03 |
| cerebral cortex | astrocyte of the cerebral cortex | 7.09 | 71.92 |
| cerebral cortex | brain pericyte | 51.49 | 160.81 |
| cerebral cortex | endothelial cell | 40.5 | 169.06 |
| cerebral cortex | macrophage | 0 | 0 |
| cerebral cortex | microglial cell | 0.79 | 13.58 |
| cerebral cortex | neuron | 0.38 | 1.04 |
| cerebral cortex | not assigned | 39.72 | 342.21 |
| cerebral cortex | oligodendrocyte | 0.16 | 1.37 |
| cerebral cortex | oligodendrocyte precursor cell | 0.21 | 1.53 |
| descending colon | Brush cell of epithelium proper of large intestine | 18.69 | 37.22 |
| descending colon | enterocyte of epithelium of large intestine | 1.53 | 13.29 |
| descending colon | enteroendocrine cell | 3.21 | 11.42 |
| descending colon | epithelial cell of large intestine | 17.9 | 40.05 |
| descending colon | large intestine goblet cell | 14.42 | 41.42 |
| descending colon | not assigned | 26.21 | 272.71 |
| diaphragm | endothelial cell | 97.72 | 344.89 |
| diaphragm | lymphocyte | 36.69 | 218.5 |
| diaphragm | macrophage | 1.35 | 5.52 |
| diaphragm | mesenchymal stem cell | 75.09 | 162.66 |
| diaphragm | not assigned | 3.8 | 21.19 |
| diaphragm | skeletal muscle satellite stem cell | 47.07 | 166.53 |
| endocrine pancreas | endothelial cell | 38.92 | 139.13 |
| endocrine pancreas | leukocyte | 0.37 | 1.5 |
| endocrine pancreas | not assigned | 36.48 | 169.84 |
| endocrine pancreas | pancreatic A cell | 196.11 | 235.54 |
| endocrine pancreas | pancreatic acinar cell | 22.7 | 45.4 |
| endocrine pancreas | pancreatic D cell | 61.55 | 103.76 |
| endocrine pancreas | pancreatic ductal cell | 2.94 | 5.97 |
| endocrine pancreas | pancreatic PP cell | 118.41 | 155.61 |
| endocrine pancreas | pancreatic stellate cell | 61.22 | 167.96 |
| endocrine pancreas | type B pancreatic cell | 459.04 | 334.63 |
| exocrine pancreas | endothelial cell | 18.22 | 40.4 |
| exocrine pancreas | leukocyte | 0 | 0 |
| exocrine pancreas | not assigned | 0.08 | 0.76 |
| exocrine pancreas | pancreatic A cell | 28.54 | 40.37 |
| exocrine pancreas | pancreatic acinar cell | 0.8 | 5.59 |
| exocrine pancreas | pancreatic D cell | 193.85 | 272.13 |
| exocrine pancreas | pancreatic ductal cell | 20.99 | 69.43 |
| exocrine pancreas | pancreatic PP cell | 8.92 | 10.86 |
| exocrine pancreas | pancreatic stellate cell | 100.59 | 140.78 |
| exocrine pancreas | type B pancreatic cell | 502.25 | 434.88 |
| gonadal fat pad | B cell | 0 | 0 |
| gonadal fat pad | endothelial cell | 17.27 | 80.09 |
| gonadal fat pad | mesenchymal stem cell of adipose | 71.39 | 112.71 |
| gonadal fat pad | myeloid cell | 0.82 | 8.29 |
| gonadal fat pad | natural killer cell | 0.1 | 0.38 |
| gonadal fat pad | not assigned | 2.44 | 26.99 |
| gonadal fat pad | T cell | 15.71 | 47.26 |
| heart | cardiac muscle cell | 3.92 | 13.58 |
| heart | not assigned | 0 | 0 |
| heart left atrium | cardiac muscle cell | 1.02 | 4.1 |
| heart left atrium | cardiac neuron | 0 | 0 |
| heart left atrium | endocardial cell | 29.49 | 88.45 |
| heart left atrium | endothelial cell | 28.4 | 108.06 |
| heart left atrium | fibroblast | 37.08 | 99.23 |
| heart left atrium | leukocyte | 0.26 | 1.35 |
| heart left atrium | myofibroblast cell | 84.05 | 178.81 |
| heart left atrium | not assigned | 0 | 0 |
| heart left atrium | smooth muscle cell | 77.57 | 208.05 |
| heart left atrium and heart right atrium | cardiac muscle cell | 4.88 | 22.37 |
| heart left atrium and heart right atrium | not assigned | 0 | 0 |
| heart left ventricle | cardiac neuron | 0 | NA |
| heart left ventricle | endocardial cell | 0 | 0 |
| heart left ventricle | endothelial cell | 21.55 | 116.32 |
| heart left ventricle | fibroblast | 107.09 | 179.78 |
| heart left ventricle | leukocyte | 0.23 | 1.22 |
| heart left ventricle | myofibroblast cell | 66.38 | 123.45 |
| heart left ventricle | not assigned | 1.94 | 20.58 |
| heart left ventricle | smooth muscle cell | 41.02 | 91.04 |
| heart right atrium | cardiac muscle cell | 74.94 | 167.58 |
| heart right atrium | cardiac neuron | 0 | 0 |
| heart right atrium | endocardial cell | 7.59 | 49.43 |
| heart right atrium | endothelial cell | 21.45 | 103.9 |
| heart right atrium | fibroblast | 76 | 148.95 |
| heart right atrium | leukocyte | 0.15 | 0.84 |
| heart right atrium | myofibroblast cell | 129.36 | 318.09 |
| heart right atrium | not assigned | 0.62 | 4.54 |
| heart right atrium | smooth muscle cell | 56.54 | 129.8 |
| heart right ventricle | cardiac muscle cell | 0 | 0 |
| heart right ventricle | cardiac neuron | 0.25 | 0.66 |
| heart right ventricle | endocardial cell | 27.24 | 92.89 |
| heart right ventricle | endothelial cell | 24.78 | 104.86 |
| heart right ventricle | fibroblast | 75.7 | 141.71 |
| heart right ventricle | leukocyte | 0.28 | 1.82 |
| heart right ventricle | myofibroblast cell | 72.69 | 153.3 |
| heart right ventricle | not assigned | 37.2 | 213.7 |
| heart right ventricle | smooth muscle cell | 27.95 | 69.62 |
| hippocampus | astrocyte of the cerebral cortex | 2.4 | 21.43 |
| hippocampus | brain pericyte | 28.15 | 60.76 |
| hippocampus | endothelial cell | 21.08 | 93.55 |
| hippocampus | macrophage | 0.16 | 0.49 |
| hippocampus | microglial cell | 1.12 | 13.95 |
| hippocampus | neuron | 0 | 0 |
| hippocampus | not assigned | 7.05 | 92.11 |
| hippocampus | oligodendrocyte | 0.33 | 3.3 |
| hippocampus | oligodendrocyte precursor cell | 0.16 | 0.81 |
| interscapular brown adipose tissue | B cell | 0.13 | 0.99 |
| interscapular brown adipose tissue | endothelial cell | 8.21 | 57.57 |
| interscapular brown adipose tissue | mesenchymal stem cell of adipose | 112.65 | 154.78 |
| interscapular brown adipose tissue | myeloid cell | 0.8 | 5.37 |
| interscapular brown adipose tissue | natural killer cell | 0 | 0 |
| interscapular brown adipose tissue | not assigned | 5.32 | 51.82 |
| interscapular brown adipose tissue | T cell | 24.47 | 208.47 |
| kidney | endothelial cell | 22.18 | 107.96 |
| kidney | epithelial cell of proximal tubule | 0.58 | 5.32 |
| kidney | kidney collecting duct epithelial cell | 0 | 0 |
| kidney | macrophage | 0.08 | 0.36 |
| kidney | natural killer cell | 0.46 | 1.82 |
| kidney | not assigned | 8.23 | 61.58 |
| limb muscle | B cell | 0.04 | 0.33 |
| limb muscle | endothelial cell | 54.49 | 222.21 |
| limb muscle | macrophage | 0.55 | 2.33 |
| limb muscle | mesenchymal stem cell | 150.14 | 248.13 |
| limb muscle | not assigned | 20.05 | 85.11 |
| limb muscle | skeletal muscle satellite cell | 100.34 | 247.78 |
| limb muscle | T cell | 19.6 | 78.69 |
| liver | B cell | 0.1 | 0.66 |
| liver | endothelial cell of hepatic sinusoid | 63.99 | 164.5 |
| liver | hepatocyte | 10.79 | 40.97 |
| liver | Kupffer cell | 19.6 | 100.81 |
| liver | natural killer cell | 4.06 | 17.77 |
| liver | not assigned | 27.52 | 199.39 |
| lung | B cell | 0.07 | 0.4 |
| lung | ciliated columnar cell of tracheobronchial tree | 37.59 | 97.46 |
| lung | classical monocyte | 0.19 | 1.3 |
| lung | epithelial cell of lung | 21.24 | 59.48 |
| lung | leukocyte | 0.23 | 1.35 |
| lung | lung endothelial cell | 32.5 | 142.77 |
| lung | monocyte | 0.26 | 2.07 |
| lung | myeloid cell | 25.08 | 124.5 |
| lung | natural killer cell | 0 | 0 |
| lung | not assigned | 40.03 | 298.74 |
| lung | stromal cell | 97.33 | 191.88 |
| lung | T cell | 0.11 | 0.78 |
| mammary gland | basal cell | 2.8 | 21.79 |
| mammary gland | endothelial cell | 51.04 | 148.31 |
| mammary gland | luminal epithelial cell of mammary gland | 12.34 | 45.42 |
| mammary gland | not assigned | 41.73 | 378.68 |
| mammary gland | stromal cell | 162.53 | 259.68 |
| mesenteric adipose tissue | B cell | 0.77 | 4.88 |
| mesenteric adipose tissue | endothelial cell | 29.51 | 115 |
| mesenteric adipose tissue | mesenchymal stem cell of adipose | 55.05 | 120.89 |
| mesenteric adipose tissue | myeloid cell | 3.65 | 40.84 |
| mesenteric adipose tissue | natural killer cell | 0.03 | 0.18 |
| mesenteric adipose tissue | not assigned | 7.68 | 67.78 |
| mesenteric adipose tissue | T cell | 0.47 | 3.48 |
| spleen | B cell | 0.21 | 3.76 |
| spleen | macrophage | 4.71 | 26.87 |
| spleen | not assigned | 0 | 0 |
| spleen | T cell | 0.26 | 1.76 |
| striatum | astrocyte of the cerebral cortex | 4.55 | 32.37 |
| striatum | Bergmann glial cell | 0 | NA |
| striatum | brain pericyte | 3.53 | 8.34 |
| striatum | endothelial cell | 32.65 | 131.91 |
| striatum | macrophage | 0.93 | 1.86 |
| striatum | microglial cell | 1.29 | 17.39 |
| striatum | neuron | 0.21 | 1.6 |
| striatum | not assigned | 0.11 | 1.44 |
| striatum | oligodendrocyte | 0.19 | 1.67 |
| striatum | oligodendrocyte precursor cell | 0.01 | 0.07 |
| subcutaneous adipose tissue | B cell | 0.09 | 0.74 |
| subcutaneous adipose tissue | endothelial cell | 30.07 | 114.23 |
| subcutaneous adipose tissue | mesenchymal stem cell of adipose | 112.46 | 172.55 |
| subcutaneous adipose tissue | myeloid cell | 0.4 | 4.35 |
| subcutaneous adipose tissue | natural killer cell | 0.2 | 0.63 |
| subcutaneous adipose tissue | not assigned | 12.09 | 55.14 |
| subcutaneous adipose tissue | T cell | 1.25 | 11.16 |
| thymus | DN1 thymic pro-T cell | 0.23 | 0.91 |
| thymus | immature T cell | 0.36 | 7.02 |
| thymus | not assigned | 12.46 | 141.9 |
| thymus | professional antigen presenting cell | 4.83 | 28.66 |
| tongue | basal cell of epidermis | 5.24 | 21.16 |
| tongue | keratinocyte | 3.14 | 15.79 |
| tongue | not assigned | 0 | 0 |
| trachea | blood cell | 25.68 | 109.63 |
| trachea | endothelial cell | 39.02 | 106.39 |
| trachea | epithelial cell | 18.21 | 94.33 |
| trachea | mesenchymal cell | 93.46 | 186.8 |
| trachea | not assigned | 0 | 0 |
| urinary bladder | bladder cell | 66.42 | 106.09 |
| urinary bladder | bladder urothelial cell | 9.89 | 29 |
| urinary bladder | not assigned | 37.16 | 274.5 |

**Supplementary Table 3: IL-1R1 expression in human liver cells (data set from Wang et al**^19^**).** Normalized expression counts were downloaded from the Single Cell Expression Atlas (https://www.ebi.ac.uk/gxa/sc/experiments/E-MTAB-10553/results/tsne) and summarized by annotated cell type (mean and SD).

| **Organism part** | **Cell type** | **Mean *IL1R1*** | **SD *IL1R1*** |
| --- | --- | --- | --- |
| liver | activated HSC | 95.77 | 172.29 |
| liver | B cell | 2.67 | 26.27 |
| liver | cholangiocyte | 23.09 | 77.17 |
| liver | cycling cell | 3.51 | 19.2 |
| liver | hepatocyte | 7.71 | 50.96 |
| liver | Kupffer cell | 8.37 | 59.65 |
| liver | liver sinusoidal endothelial cell | 154.79 | 272.3 |
| liver | mesothelial cell | 82.02 | NA |
| liver | monocyte-derived macrophage | 19.07 | 69.52 |
| liver | natural killer cell | 2.97 | 38.59 |
| liver | not assigned | 24.41 | 129.86 |
| liver | plasma cell | 3.77 | 25.41 |
| liver | quiescent HSC | 27.72 | 48.01 |
| liver | T cell | 4.72 | 41.44 |
| liver | vascular endothelial cell 1 | 52.67 | 124.06 |
| liver | vascular endothelial cell 2 | 19.32 | 36.31 |

**Supplementary Table 4: IL-1R1 expression in human liver cells (data set from MacParland et al ^20^).** Normalized expression counts were downloaded from the Single Cell Expression Atlas (https://www.ebi.ac.uk/gxa/sc/experiments/E-HCAD-9/results/tsne) and summarized by annotated cell type (mean and SD).

| **Organism part** | **Cell type** | **Mean *IL1R1*** | **SD *IL1R1*** |
| --- | --- | --- | --- |
| liver | alpha-beta T cell | 2.84 | 31.59 |
| liver | central venous liver sinusoidal endothelial cell | 181.02 | 301.29 |
| liver | cholangiocyte | 19.31 | 76.85 |
| liver | erythroid cell | 5.76 | 28.25 |
| liver | gamma-delta T cell | 1.75 | 24.47 |
| liver | hepatic stellate cell | 155.57 | 215.76 |
| liver | hepatocyte | 19.43 | 75.01 |
| liver | inflammatory macrophage | 10.19 | 74.68 |
| liver | mature B cell | 0 | 0 |
| liver | NK-like cell | 1.69 | 29.11 |
| liver | non-inflammatory macrophage | 4.2 | 47.7 |
| liver | not assigned | 36.04 | 210.47 |
| liver | periportal liver sinusoidal endothelial cell | 112.07 | 227.3 |
| liver | plasma cell | 0.67 | 9.11 |
| liver | portal liver sinusoidal endothelial cell | 421.81 | 406.8 |

**Supplementary Table 5: Mouse forward and reverse primers used for qRT-PCR.**

| **Gene** | **Protein** | **Forward primer** | **Reverse primer** |
| --- | --- | --- | --- |
| *Acaca* | ACC | AAC ATC CCC ACG CTA AAC AG | CTG ACA AGG TGG CGT GAA G |
| *Adgre1* | F4/80 | CTT TGG CTA TGG GCT TCT AGT C | GCA AGG AGG ACA GAG TTT ATC GTG |
| *Apoc2* | APOC2 | GAG CCA GGA TAG TCC CTT CC | AAA ATG CCT GCG TAA GTG CT |
| *Arg1* | ARG1 | CTC CAA GCC AAA GTC CTT AGA G | AGG AGC TGT CAT TAG GGA CAT C |
| *Ccl2* | CCL2 | CTT CTG GGC CTG CTG TTC A | CCA GCC TAC TCA TTG GGA TCA |
| *Ccr2* | CCR2 | AGA GGT CTC GGT TGG GTT GT | ATC ATA ACG TTC TGG GCA CC |
| *Cd36* | FAT/CD36 | AAT CCT CTC CCT CTC TGG TGT C | CAT GGC GAG GAA CAG AAC AT |
| *Ces1d* | CES1 | CCT ACC CTC CTT TGT GCT AC | CGG CTG TGT TCA TCC TCT |
| *Cpt1a* | CPT1 | AGT GGC CTC ACA GAC TCC AG | GCC ATG TTG TAC AGC TTC C |
| *Cxcl1* | CXCL-1 | ATC CAG AGC TTG AAG GGG TTG | GTC TGT CTT CTT TCT CCG TTA CTT |
| *Cxcl2* | CXCL-2 | CTC TCA AGG GCG GTC AAA AAG TT | TCA GAC AGC GAG GCA CAT CAG GTA |
| *Fasn* | FAS | CCC TTG ATG AAG AGG GAT CA | GAA CAA GGC GTT AGG GTT GA |
| *Fbp1* | FBP1 | ATG AGG GTT ATG CCA AGG ACT TT | CCA TCC GGA GGG AAC TTT TT |
| *G6pc* | G6Pase | TCC TGG GAC AGA CAC ACA AG | CAA CTT TAA TAT ACG CTA TTG G |
| *Hmox1* | HO-1 | CCT CAC TGG CAG GAA ATC ATC | CCT CGT GGA GAC GCT TTA CAT A |
| *Il1a* | IL-1α | CAA ACT GAT GAA GCT CGT CA | TCT CCT TGA GCG CTC ACG AA |
| *Il1b* | IL-1β | TCT TTG AAG TTG ACG GAC CC | TGA GTG ATA CTG CCT GCC TG |
| *Il1rn* | IL-1Ra | GGG ATA CTA ACC AGA AGA CC | GAC AGG CAC AGC TTG CCC CC |
| *Il6* | IL-6 | AGT TGC CTT CTT GGG ACT GA | TTC TGC AAG TGC ATC ATC GT |
| *Irs1* | IRS-1 | AGC GAG CTC GAG CAT GGC GAG CCC TC | ATC GTC GAC TCG AGA TCT CCG AGT CA |
| *Irs2* | IRS-2 | CTC TGA CTA TAT GAA CCT G | ACC TTC TGG CTT TGG AGG TG |
| *Lep* | Leptin | TGC TGC AGA TAG CCA ATG AC | GAG TAG AGT GAG GCT TCC AGG A |
| *Lpl* | LPL | CGA GAG CGA GAA CAT TCC CT | TGT CCA CCT CCG TGT AAA TCA A |
| *Mrc1* | CD206 | TGG GGT GCT GAC GAG CCG AA | ACC AGG GAG GCA CCC ATT CGA |
| *Mlxipl* | ChREBP | GCA TCC TCA TCC GAC CTT TA | GAT GCT TGT GGA AGT GCT GA |
| *Mttp* | MTP | TGA GCG GCT ATA CAA GCT CAC | CTG GAA GAT GCT CTT CTC GC |
| *Nr1h4* | FXR-α | CCC AGA GAA GAA CCG AGT T | TAG ATG CCA GGA GAA TAC CAG |
| *Nrf1* | NRF-1 | AGC ACG GAG TGA CCC AAA C | TGT ACG TGG CTA CAT GGA CCT |
| *Pck1* | PEPCK | CTT CTC TGC CAA GGT CAT CC | TTT TGG GGA TGG GCA C |
| *Pcx* | PC | GAT GAC CTC ACA GCC AAG CA | GGG TAC CTC TGT GTC CAA AGG A |
| *Ppara* | PPAR-α | CAG TGG GGA GAG AGG ACA GA | AGT TCG GGA ACA AGA CGT TG |
| *Pparg* | PPAR-γ | GAT GGA AGA CCA CTC GCA TT | AAC CAT TGG GTC AGC TCT TG |
| *Ppargc1a* | PGC-1α | CGG AAA TCA TAT CCA ACC AG | TGA GGA CCG CTA GCA AGT TTG |
| *Scd1* | SCD1 | GCT GGG CAG GAA CTA GTG AG | GAA GGC ATG GAA GGT TCA AA |
| *Sirt1* | SIRT1 | CGG CTA CCG AGG TCC ATA TAC | ACA ATC TGC CAC AGC GTC AT |
| *Slc2a4* | GLUT4 | CTG TGC CAT CTT GAT GAC CGT G | GTT GGA GAA ACC AGC GAC AGC |
| *Srebf1* | SREBP-1c | ATC TCC TAG AGC GAG CGT TG | TAT TTA GCA ACT GCA GAT ATC CAA G |
| *Tfam* | TFAM | AAG GGA ATG GGA AAG GTA GA | AAC AGG ACA TGG AAA GCA GAT |
| *Tnf* | TNF-α | GAA GTT CCC AAA TGG CCT CC | GTG AGG GTC TGG GCC ATA GA |

**Supplementary Table 6: Human forward and reverse primers used for qRT-PCR.**

| **Gene** | **Protein** | **Forward primer** | **Reverse primer** |
| --- | --- | --- | --- |
| *CPT1A* | CPT1 | ATG CGC TAC TCC CTG AAA GTG | GTG GCA CGA CTC ATC TTG C |
| *CXCL8* | IL-8 | TTG GCA GCC TTC CTG ATT | AAC TTC TCC ACA ACC CTC TG |
| *GAPDH* | GAPDH | CTC TGC TCC TCC TGT TCG AC | ACG ACC AAA TCC GTT GAC TC |
| *IL1R1* | IL-1R1 | AAG GTG GAG GAT TCA GGA CAT | AGC CTA TCT TTG ACT CCA CTA |
| *IL1RAP* | IL-1RAcP | AGA ACC GCA TTA GTA AGG AGA AA | CTT TAC AGT CAG AGT CCT GGT GAG |
| *NR1H4* | FXR-α | TGT GAG GGG TGT AAA GGT TTC T | GCC TGT ATA CAT ACA TTC AGC CA |
| *PPARA* | PPAR-α | TCA CAA GTG CCT TTC TGT CG | TCT TGG CAT TCG TCC AAA A |
| *PPARGC1A* | PGC-1α | CCA AAC CAA CAA CTT TAT CTC TTC C | CAC ACT TAA GGT GCG TTC AAT AGT C |
| *SREBF1* | SREBP-1c | GGA GGG GTA GGG CCA ACG | AGG GGT GGA GCT GAA CTG |

**Supplementary Tables – Results:**

**Supplementary Table 7: Significantly differentially expressed genes from comparison of naïve, male *Il1r1*^Hep-/-^ vs. WT mice at 10 weeks of age (n=6 mice/genotype).** The table shows the mean normalized counts (baseMean), expression change (log2FoldChange), and statistical significance (padj = FDR-adjusted p-value).

| **Gene_name** | **baseMean** | **log2FoldChange** | **padj** |
| --- | --- | --- | --- |
| *Socs3* | 273.74 | 1.065 | 0.000939 |
| *Trabd* | 2044.64 | -0.348 | 0.009257 |
| *Il1r1* | 1929.39 | -1.001 | 0.019099 |
| *Creld2* | 1196.77 | -1.030 | 0.026727 |
| *Edem1* | 6551.15 | -0.272 | 0.026727 |
| *Ifit1* | 241.83 | 0.731 | 0.026727 |
| *Alg12* | 220.98 | -0.637 | 0.026727 |
| *BC024978* | 323.94 | -0.415 | 0.026727 |
| *Cad* | 489.89 | -0.672 | 0.045937 |
| *Arhgap6* | 165.01 | 0.600 | 0.045937 |
